# Novel Fluorinated Chlorhexidine Analogues Overcome Resistance in Gram-Negative Bacteria

**DOI:** 10.1021/acsomega.5c11738

**Published:** 2026-04-28

**Authors:** Yunxiao Li, Maida Jajja, Jiajing Lu, Nasima S. Chowdhury, Charlotte Hind, J. Mark Sutton, Khondaker Miraz Rahman

**Affiliations:** † School of Cancer and Pharmaceutical Sciences, 4616 King’s College London, Franklin-Wilkins Building, 150 Stamford Street, London SE1 9NH, U.K.; ‡ Countermeasures Development, Evaluation and Preparedness, Public Health Microbiology, UK Health Security Agency, Manor Farm Road, Porton Down, Salisbury SP4 0JG, U.K.

## Abstract

Antimicrobial resistance (AMR) is a critical global health challenge that compromises the effectiveness of widely used biocides such as **chlorhexidine** (CHX). The emergence of CHX-resistant bacterial strains, often mediated by efflux mechanisms and membrane adaptations, necessitates the development of new chemical entities capable of overcoming these resistance pathways. Here, we report the rational design, synthesis, and biological evaluation of structurally modified CHX analogues. These analogues were designed by substituting the terminal chlorophenyl rings of CHX with strategically selected aromatic groups bearing fluorine and methyl substituents to enhance membrane permeability, modulate physicochemical properties, and reduce efflux susceptibility. A library of 13 compounds was prepared using biscyanoguanidine chemistry and aniline-based substitutions via microwave-assisted synthesis, and was fully characterized by LC–MS, HRMS, and NMR. Antibacterial activity was assessed using minimum inhibitory concentration (MIC) and minimum bactericidal concentration (MBC) assays against a panel of Gram-positive and Gram-negative bacteria, including CHX-resistant *Klebsiella pneumoniae* and *Pseudomonas aeruginosa* strains harboring *smvR*, *phoQ*, and *pmrB* mutations. Selected analogues, particularly compounds **8** and **11**, demonstrated potent antibacterial activity. Compound **11** showed MIC values predominantly in the range of 4–8 μg/mL across wild-type strains and retained activity in resistant isolates, with MIC values of 4–8 μg/mL in *P. aeruginosa* and 64 μg/mL in CHX-resistant *K. pneumoniae*. MBC values were generally comparable to MIC values for the fluorinated analogues, consistent with a biocidal mode of action, while chlorhexidine showed elevated MBC values in resistant strains. Molecular modeling suggests that these compounds form favorable interactions within hydrophobic regions of the SmvA efflux pump in *K. pneumoniae*, potentially reducing efflux susceptibility. Structure–activity relationship analysis highlights the importance of combining fluorine and methyl substituents to optimize physicochemical properties associated with antibacterial activity and resistance bypass. Collectively, these findings establish a foundation for the development of next-generation CHX-based biocides with improved efficacy against multidrug-resistant pathogens and support further translational evaluation.

## Graphical abstract

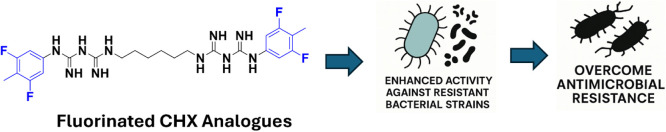

## Introduction

The continued rise of antimicrobial resistance (AMR) poses a profound and urgent threat to public health worldwide. With an estimated 4.95 million deaths associated with AMR in 2019 and projections reaching up to 10 million deaths annually by 2050, the need for novel antimicrobial strategies is undeniable.^1–3^ Antimicrobial resistance undermines decades of progress in the treatment of infectious diseases and presents significant clinical and economic burdens.^4–7^ While antibiotics remain the cornerstone of bacterial infection management, their overuse and misuse, alongside bacteria’s inherent capacity for genetic adaptation, have fueled the rapid spread of multidrug-resistant (MDR) pathogens.^5,8^

Biocides, such as **chlorhexidine** (CHX), serve as vital tools for infection control, especially in hospital and clinical settings.^9^ CHX is a widely used biguanide-based antiseptic characterized by its broad-spectrum activity, low toxicity, and versatility in applications ranging from oral care to surgical site disinfection.^10,11^ Its molecular structure consists of two para-chlorophenyl end groups and two biguanide moieties, connected by a flexible hexamethylene linker. This symmetrical dicationic structure renders chlorhexidine positively charged under physiological conditions.^12,13^ Its mechanism of action primarily involves disrupting the bacterial cell membrane, leading to leakage of cytoplasmic contents and eventual cell death.^14,15^ Although **chlorhexidine** is effective against most pathogenic microorganisms, higher concentrations are required to inhibit or kill Gram-negative bacteria effectively and it has minimal or no activity against bacterial spores.^16–18^ Meanwhile, overuse of CHX has driven the emergence of resistant bacterial strains, particularly those with upregulated efflux pumps like qacA/B, smvR, and RND family proteins.^14,19–22^ Resistance to CHX is increasingly evident in intensive care units^23^ and long-term care facilities, where CHX is used extensively for daily bathing, catheter care, and preoperative skin preparation, raising concerns about the reliability of routine decontamination protocols.^14^ Similar trends are observed in dental settings, where rising tolerance among oral streptococci may compromise the effectiveness of CHX mouthwashes and gels and alter oral microbial ecology.^24,25^ The frequent and sustained exposure to CHX across these environments not only selects for biocide-tolerant strains but may also facilitate broader cross-resistance^26^ and environmental persistence.^27^ Concerns regarding CHX’s cytotoxic effects on epithelial and fibroblast cells further complicate decisions to increase concentration or duration of use as a countermeasure to resistance.^28^ These developments highlight the clinical urgency of understanding and mitigating emerging CHX resistance.^26,29,30^

Efflux pumps are a key mechanism of bacterial resistance against CHX. These membrane-bound proteins expel a variety of antimicrobial agents, including biocides, out of bacterial cells, thereby reducing intracellular concentrations and limiting efficacy.^19,20^ Gram-negative bacteria, such as *Pseudomonas aeruginosa* and *Klebsiella pneumoniae*, are especially notorious for their intrinsic and acquired efflux mechanisms, rendering them less susceptible to CHX.^20,31^ Structural modifications of CHX, specifically targeting its aromatic end groups and linker chains, offer a rational approach to circumvent these resistance mechanisms.

This study aims to overcome these resistance mechanisms through rational design of CHX analogues with enhanced efficacy and resistance-bypassing potential. We hypothesize that substituting the terminal chlorophenyl rings of CHX with strategically selected aromatic groups, particularly those bearing fluorine and methyl substituents at defined positions, can improve antimicrobial potency and reduce efflux susceptibility, and overcome chlorhexidine resistance mechanism in Gram-negative bacteria. Fluorinated aromatic rings are known to enhance membrane permeability^32^ and increase lipophilicity which can facilitate penetration across the outer membrane barrier of Gram-negative bacteria, while also reducing recognition by efflux systems.^33^ In addition, fluorine substitution can influence molecular conformation and dipole characteristics, which may reduce binding affinity to efflux transporters. Methyl groups can further modulate hydrophobicity and steric interactions,^34^ potentially enhancing membrane association and limiting efflux pump engagement. Building on this rationale, we synthesized a series of CHX analogues via biscyanoguanidine chemistry, introducing mono-, di-, and trisubstituted phenyl rings with varied fluorine and alkyl substitution patterns.

The objective of this study is to evaluate the antimicrobial activity of these analogues against a panel of Gram-positive and Gram-negative bacterial strains, including CHX-resistant clinical isolates with known resistance mutations (*smvR, phoQ, pmrB, fleQ, ampDH2*). Minimum Inhibitory Concentration (MIC) values were determined to assess efficacy, and structure–activity relationships (SAR) were analyzed to identify substitution patterns that confer optimal activity. This work aims to establish a clear SAR framework to guide future development of next-generation CHX-based antimicrobials with improved potency and resistance evasion.

## Materials and Methods

### Chemistry

#### General Chemistry

All reactions were carried out in oven-dried glassware and all reagents were obtained commercially either from Fluorochem, Fisher Scientific or Sigma-Aldrich Ltd.

The reactions were monitored, and products were identified using Liquid Chromatography–Mass Spectrometry (LC-MS). LC-MS system used is a Waters Alliance 2695 system with an elution in gradient. Low-resolution mass spectra were analyzed and recorded on a Waters QZ instrument using electrospray ionization (ESI) and coupled to a High-performance liquid chromatography (HPLC) system. Selected mass-to-charge ratio peaks (*m*/*z*) are quoted in Daltons. HPLC grades solvents were used for mobile phase and a Phenomenex Monolithic C18 50 × 4.60 mm column was used for stationary phase. Detect method used UV detection performed on a Waters 2996 photo array detector. Purity is calculated using the HPLC-LCMS peak areas.

Purification of target compounds was performed using Preparative High Performance Liquid Chromatography (Prep HPLC) on an Agilent 1290 Infinity II Preparative LCMSD System. A Phenomenex Luna 5 μm C18(2) 100 Å LC column (100 × 21.2 mm) was used, with water (0.1% formic acid) and acetonitrile as mobile phases at a flow rate of 20 mL/min. The gradient conditions were adjusted for each specific product. Solvents were evaporated using a freeze-dryer at −50 °C for 48–72 h to obtain the pure product.

The pure compounds were characterized by Nuclear Magnetic Resonance (NMR), including ^1^H NMR, ^13^C NMR, COSY, HSQC, and HMBC, using a Bruker Spectrospin Spectrometer (Bruker Corp., MA, USA) equipped with a SampleXpress autosampler system. The residual solvent peak was used as the internal standard for chemical shifts, with chemical shifts (δ) and coupling constants (J) reported in parts per million (ppm) and Hertz (Hz), respectively. Assignment of ^1^H and ^13^C NMR spectra were made using the aid of TopSpin 3.5 software from Bruker or MestReNova from Mestrelab Research.

High Resolution Mass Spectra (HRMS) were obtained using a Thermo Navigator mass spectrometer coupled with liquid chromatography (LC) using electrospray ionization and time-of-flight mass spectrometry.

### Synthesis of Chlorhexidine Analogues

All analogues were synthesized in a one-step process, the substituted aromatic amine (Ar–NH_2_) connected to a bis-biguanide compound bearing a flexible hexamethylene linker by a condensation reaction to form the symmetrical biguanide compounds (Scheme 1).^35^ Key substituted groups included fluorine, methyl, methoxy, and diamino derivatives.

**Scheme 1:**
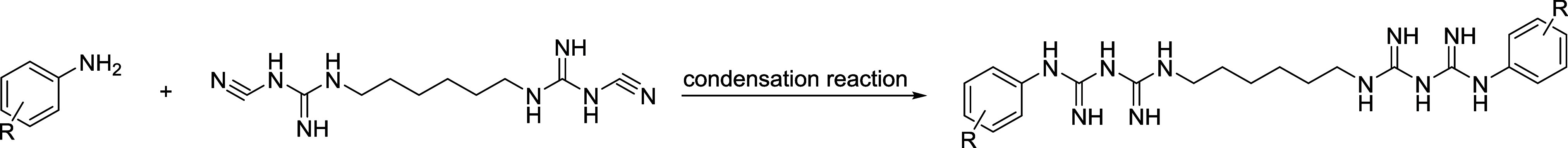
General Synthetic Routes for Compounds Where Substituted Aromatic Amines Were Connected to the Linker^a^

a Conditions: HCl, 2-ethoxyethanol 180 °C or water 150 °C, microwave.

In an oven-dried 20 mL microwave tube, *N*,*N*′′′-1,6-Hexanediylbis­(*N*′-cyanoguanidine) (1.0 equiv), 2-ethoxyethanol or water and substituted aniline (5.0 or 10.0 equiv), and hydrochloric acid (1 or 2 drops) were added sequentially. The mixture was then heated under microwave at 180 °C (2-ethoxyethanol as solvent) or 150 °C (water as solvent) for 50 min. Crude products were subjected to flash chromatography and further purified via reverse-phase preparative HPLC. Structural identification was achieved through LC-MS and HRMS analysis. NMR characterization (1H, 13C, and HSQC) confirmed compound integrity and substitution pattern.

*N-Phenyl-1-[N*′*-(6-{N-[(N*′*-phenylcarbamimidamido methanimidoyl]­amino}­hexyl*)­carbamimidamido*]­methanimidamide* **(1)** was synthesized from *N*,*N*′′′-1,6-Hexanediylbis­(*N*′-cyanoguanidine) (1.0 equiv) and aniline (5.0 equiv) in deionized water with hydrochloric acid (1 drop). The mixture was heated under microwave irradiation at 150 °C for 50 min. The crude compound was purified by flash chromatography on the Biotage system using water/acetonitrile as the eluent to afford **compound 1** as white solid (yield 77.2%); ^1^H NMR (400 MHz, MeOD) δ 7.27–7.17 (m, 8H), 7.04–6.98 (m, 2H), 3.08 (t, J = 7.2 Hz, 4H), 1.44 (s, 4H), 1.25 (s, 4H); ^13^C NMR (101 MHz, MeOD) δ 168.75, 128.57, 124.30, 121.87, 41.35, 29.56–28.16 (m), 26.09.

*N-(4-Fluorophenyl)-1-{N*′*-[6-(N-{[N*′*-(4-fluorophenyl*)­carbamimidamido*]­methanimidoyl}­amino*)­hexyl*]­carbamimidamido}­methanimidamide* **(2)** was synthesized from *N*,*N*′′′-1,6-Hexanediylbis­(*N*′-cyanoguanidine) (1.0 equiv) and 4-fluoroaniline (5.0 equiv) in 2-ethoxyethanol with hydrochloric acid (1 drop). The mixture was heated under microwave irradiation at 180 °C for 50 min. The crude compound was purified by the preparative HPLC system using water/acetonitrile with 0.1% Formic acid as the eluent to afford **compound 2** as light yellow solid (yield 20.8%); ^1^H NMR (400 MHz, MeOD) δ 8.39 (s, 1H), 7.30–7.22 (m, 4H), 6.95 (t, J = 8.7 Hz, 4H), 3.07 (t, J = 7.1 Hz, 4H), 1.43 (s, 4H), 1.25 (q, J = 6.8 Hz, 4H); ^13^C NMR (101 MHz, MeOD) δ 127.71 (d, J = 8.8 Hz), 124.18, 115.05 (d, J = 22.8 Hz), 41.33, 28.96, 26.10.

*N-(4-Methylphenyl)-1-{N*′*-[6-(N-{[N*′*-(4-methylphenyl*)­carbamimidamido*]­methanimidoyl}­amino*)­hexyl*]­carbamimidamido}­methanimidamide* **(3)** was synthesized from *N*,*N*′′′-1,6-Hexanediylbis­(*N*′-cyanoguanidine) (1.0 equiv) and p-toluidine (5.0 equiv) in deionized water with hydrochloric acid (2 drops). The mixture was heated under microwave irradiation at 150 °C for 50 min. The crude compound was purified by the preparative HPLC system using water/acetonitrile with 0.1% Formic acid as the eluent to afford **compound 3** as yellow solid (yield 13.1%); ^1^H NMR (400 MHz, MeOD) δ 7.14–7.08 (m, 4H), 7.05 (d, J = 8.4 Hz, 4H), 3.08 (t, J = 7.1 Hz, 4H), 2.20 (s, 6H), 1.54–1.32 (m, 4H), 1.31–1.17 (m, 4H); ^13^C NMR (101 MHz, MeOD) δ 134.81, 129.22, 122.52, 41.40, 28.86, 26.10, 19.52.

*N-(4-Methoxyphenyl)-1-{N*′*-[6-(N-{[N*′*-(4-methoxyphenyl*)­carbamimidamido*]­methanimidoyl}­amino*)­hexyl*]­carbamimidamido}­methanimidamide* **(4)** was synthesized from *N*,*N*′′′-1,6-Hexanediylbis­(*N*′-cyanoguanidine) (1.0 equiv) and 4-methoxyaniline (5.0 equiv) in 2-ethoxyethanol with hydrochloric acid (2 drops). The mixture was heated under microwave irradiation at 180 °C for 50 min. The crude compound was purified by the preparative HPLC system using water/acetonitrile with 0.1% Formic acid as the eluent to afford **compound 4** as light yellow solid (yield 63%); ^1^H NMR (400 MHz, MeOD) δ 7.19–7.05 (m, 4H), 6.85–6.75 (m, 4H), 3.67 (s, 6H), 3.07 (t, J = 7.1 Hz, 4H), 1.46–1.38 (m, 4H), 1.24 (d, J = 6.4 Hz, 4H); ^13^C NMR (101 MHz, MeOD) δ 130.29, 127.24 (d, J = 34.0 Hz), 124.68, 113.91, 41.26, 28.87, 26.12.

*N-(4-Fluoro-3-methylphenyl)-1-{N*′*-[6-(N-{[N*′*-(4-fluoro-3-methylphenyl*)­carbamimidamido*]­methanimidoyl}­amino*)­hexyl*]­carbamimidamido}­methanimidamide* **(5)** was synthesized from *N*,*N*′′′-1,6-Hexanediylbis­(*N*′-cyanoguanidine) (1.0 equiv) and 4-fluoro-3-methylaniline (10.0 equiv) in 2-ethoxyethanol with hydrochloric acid (2 drops). The mixture was heated under microwave irradiation at 180 °C for 50 min. The crude compound was purified by the preparative HPLC system using water/acetonitrile with 0.1% Formic acid as the eluent to afford **compound 5** as light yellow solid (yield 33.8%); ^1^H NMR (400 MHz, MeOD) δ 7.14–7.00 (m, 4H), 6.87 (t, J = 9.0 Hz, 2H), 3.06 (h, J = 6.8 Hz, 4H), 2.14 (d, J = 2.0 Hz, 6H), 1.43 (p, J = 6.1 Hz, 4H), 1.24 (s, 4H); ^13^C NMR (101 MHz, MeOD) δ 126.96–123.59 (m), 121.63, 114.68 (d, J = 23.8 Hz), 41.29, 28.91, 26.05 (d, J = 15.3 Hz), 13.19 (d, J = 3.4 Hz).

*N-(3-Fluorophenyl)-1-{N*′*-[6-(N-{[N*′*-(3-fluorophenyl*)­carbamimidamido*]­methanimidoyl}­amino*)­hexyl*]­carbamimidamido}­methanimidamide* **(6)** was synthesized from *N*,*N*′′′-1,6-Hexanediylbis­(*N*′-cyanoguanidine) (1.0 equiv) and 3-fluoroaniline (5.0 equiv) in 2-ethoxyethanol with hydrochloric acid (2 drops). The mixture was heated under microwave irradiation at 180 °C for 50 min. The crude compound was purified by the preparative HPLC system using water/acetonitrile with 0.1% Formic acid as the eluent to afford **compound 6** as light yellow solid (yield 58.8%); ^1^H NMR (400 MHz, MeOD) δ 7.19 (ddd, J = 14.8, 10.8, 8.6 Hz, 4H), 7.05–6.85 (m, 2H), 6.70 (t, J = 8.0 Hz, 2H), 3.10 (dp, J = 15.6, 7.8 Hz, 4H), 1.47 (s, 4H), 1.26 (d, J = 14.2 Hz, 4H); ^13^C NMR (101 MHz, MeOD) δ 116.43, 109.98, 107.94, 41.37, 28.64 (d, J = 118.3 Hz), 26.10.

*N-(3,4-Difluorophenyl)-1-{N*′*-[6-(N-{[N*′*-(3,4-difluorophenyl*)­carbamimidamido*]­methanimidoyl}­amino*)­hexyl*]­carbamimidamido}­methanimidamide* **(7)** was synthesized from *N*,*N*′′′-1,6-Hexanediylbis­(*N*′-cyanoguanidine) (1.0 equiv) and 3,4-difluoroaniline (10.0 equiv) in 2-ethoxyethanol with hydrochloric acid (2 drops). The mixture was heated under microwave irradiation at 180 °C for 50 min. The crude compound was purified by the preparative HPLC system using water/acetonitrile with 0.1% Formic acid as the eluent to afford **compound 7** as white solid (yield 34.3%); ^1^H NMR (400 MHz, MeOD) δ 7.37 (dd, J = 13.1, 7.4 Hz, 2H), 7.09 (dd, J = 13.4, 5.5 Hz, 2H), 7.02–6.87 (m, 2H), 3.10 (t, J = 7.1 Hz, 4H), 1.47 (q, J = 16.5 Hz, 4H), 1.28 (s, 4H); ^13^C NMR (101 MHz, MeOD) δ 117.53, 116.73 (d, J = 18.2 Hz), 110.76, 41.28, 29.24, 26.12.

*N-(3-Fluoro-4-methylphenyl)-1-{N*′*-[6-(N-{[N*′*-(3-fluoro-4-methylphenyl*)­carbamimidamido*]­methanimidoyl}­amino*)­hexyl*]­carbamimidamido}­methanimidamide* **(8)** was synthesized from *N*,*N*′′′-1,6-Hexanediylbis­(*N*′-cyanoguanidine) (1.0 equiv) and 3-fluoro-4-methylaniline (10.0 equiv) in 2-ethoxyethanol with hydrochloric acid (2 drops). The mixture was heated under microwave irradiation at 180 °C for 50 min. The crude compound was purified by flash chromatography on the Biotage system using water/acetonitrile as the eluent to afford **compound 8** as yellow solid (yield 62.9%); ^1^H NMR (400 MHz, MeOD) δ 7.16–7.07 (m, 2H), 7.10–6.95 (m, 2H), 6.87 (dd, J = 8.2, 2.2 Hz, 2H), 3.14–3.06 (m, 4H), 2.10 (t, J = 3.2 Hz, 6H), 1.47 (s, 4H), 1.27 (s, 4H); ^13^C NMR (101 MHz, MeOD) δ 131.02 (d, J = 6.3 Hz), 116.76, 108.22, 41.36, 29.14, 26.10, 12.54 (d, J = 3.4 Hz).

*N-(3-Fluoro-4-methoxyphenyl)-1-{N*′*-[6-(N-{[N*′*-(3-fluoro-4-methoxyphenyl*)­carbamimidamido*]­methanimidoyl}­amino*)­hexyl*]­carbamimidamido}­methanimidamide* **(9)** was synthesized from *N*,*N*′′′-1,6-Hexanediylbis­(*N*′-cyanoguanidine) (1.0 equiv) and 3-fluoro-4-methoxyaniline (5.0 equiv) in 2-ethoxyethanol with hydrochloric acid (4 drops). The mixture was heated under microwave irradiation at 180 °C for 50 min. The crude compound was purified by the preparative HPLC system using water/acetonitrile with 0.1% Formic acid as the eluent to afford **compound 9** as amber solid (yield 28.3%); ^1^H NMR (400 MHz, MeOD) δ 7.15 (ddd, J = 12.8, 4.7, 2.2 Hz, 2H), 7.05–6.82 (m, 4H), 3.75 (d, J = 4.9 Hz, 6H), 3.11–2.97 (m, 4H), 1.44 (s, 4H), 1.32–1.20 (m, 4H); ^13^C NMR (101 MHz, MeOD) δ 118.09, 113.63, 111.44–110.37 (m), 55.66, 41.12 (d, J = 45.3 Hz), 28.88, 26.48–25.50 (m).

*N-(4-Fluoro-3-methoxyphenyl)-1-{N*′*-[6-(N-{[N*′*-(4-fluoro-3-methoxyphenyl*)­carbamimidamido*]­methanimidoyl}­amino*)*hexyl]­carbamimidamido}­methanimidamide* **(10)** was synthesized from *N*,*N*′′′-1,6-Hexanediylbis­(*N*′-cyanoguanidine) (1.0 equiv) and 4-fluoro-3-methoxyaniline (10.0 equiv) in 2-ethoxyethanol with hydrochloric acid (2 drops). The mixture was heated under microwave irradiation at 180 °C for 50 min. The crude compound was purified by the preparative HPLC system using water/acetonitrile with 0.1% Formic acid as the eluent to afford **compound 10** as yellow solid (yield 29.9%); ^1^H NMR (400 MHz, MeOD) δ 7.10–7.01 (m, 2H), 6.96–6.85 (m, 2H), 6.81–6.70 (m, 2H), 3.75 (d, J = 3.5 Hz, 6H), 3.07 (t, J = 7.0 Hz, 4H), 1.43 (s, 4H), 1.24 (s, 4H). ^13^C NMR (101 MHz, MeOD) δ 167.04, 159.87, 147.59, 117.96, 116.41, 115.45, 115.25, 114.45, 108.68, 55.52, 55.42, 48.10, 41.35, 28.97, 26.12.

*N-(3,5-Fluoro-4-methylphenyl)-1-{N*′*-[6-(N-{[N*′*-(3,5-fluoro-4-methylphenyl*)­carbamimidamido*]­methanimidoyl}­amino*)­hexyl*]­carbamimidamido}­methanimidamide* **(11)** was synthesized from *N*,*N*′′′-1,6-Hexanediylbis­(*N*′-cyanoguanidine) (1.0 equiv) and 3,5-Difluoro-4-methylaniline (5.0 equiv) in 2-ethoxyethanol with hydrochloric acid (1 drop). The mixture was heated under microwave irradiation at 80 °C for 5 h. The crude compound was purified by the preparative HPLC system using water/acetonitrile with 0.1% Formic acid as the eluent to afford **compound 11** as yellow solid (yield 4.3%); ^1^H NMR (400 MHz, MeOD) 8.45 ppm (s, 1H), 6.92 ppm (d, J = 8.8 Hz, 4H), 3.11 ppm (m, 4H), 2.01 ppm (d, J = 1.7 Hz, 6H), 1.49 ppm (s, 4H), 1.29 ppm (d, J = 12.4 Hz, 4H). ^13^C NMR (101 MHz, MeOD) δ 168.72, 162.54, 162.43, 160.13, 160.02, 48.31, 48.10, 47.88, 46.42, 41.32, 29.33, 26.10, 25.97, 7.79, 5.16, 5.13, 5.09.

*N-(2,3-Fluoro-4-methylphenyl)-1-{N*′*-[6-(N-{[N*′*-(2,3-fluoro-4-methylphenyl*)­carbamimidamido*]­methanimidoyl}­amino*)­hexyl*]­carbamimidamido}­methanimidamide* **(12)** was synthesized from *N*,*N*′′′-1,6-Hexanediylbis­(*N*′-cyanoguanidine) (1.0 equiv) and 2,3-difluoro-4-methylaniline (5.0 equiv) in 2-ethoxyethanol with hydrochloric acid (2 drops). The mixture was heated under microwave irradiation at 50 °C for 5 h. The crude compound was purified by the preparative HPLC system using water/acetonitrile with 0.1% Formic acid as the eluent to afford **compound 12** as white solid (yield 14.6%); ^1^H NMR (400 MHz, MeOD) 8.45 ppm (s, 1H), 6.92 ppm (d, J = 8.8 Hz, 4H), 3.11 ppm (m, 4H), 2.01 ppm (d, J = 1.7 Hz, 6H), 1.49 ppm (s, 4H), 1.29 ppm (d, J = 12.4 Hz, 4H); ^13^C NMR (101 MHz, DMSO) 150.45, 150.33, 148.04, 147.92, 125.33, 120.68, 46.00, 41.33, 40.91, 31.16, 26.27, 14.11, 14.09, 14.06, 11.43.

*N-(3-Fluoro-5-amino-4-methylphenyl)-1-{N*′*-[6-(N-{[N*′*-(3-fluoro-5-amino-4-methylphenyl*)­carbamimidamido*]­methanimidoyl}­amino*)­hexyl*]­carbamimidamido}­methanimidamide* **(13)** was synthesized from *N*,*N*′′′-1,6-Hexanediylbis­(*N*′-cyanoguanidine) (1.0 equiv) and 4-Fluoro-5-methylbenzene-1,2-diamine (5.0 equiv) in 2-ethoxyethanol with hydrochloric acid (1 drop). The mixture was heated under microwave irradiation at 80 °C for 5 h. The crude compound was purified by the preparative HPLC system using water/acetonitrile with 0.1% Formic acid as the eluent to afford **compound 13** as pink red solid (yield 4.0%); ^1^H NMR (400 MHz, MeOD) 7.48 ppm (d, J = 8.9 Hz, 2H), 7.27 ppm (s, 2H), 7.13 ppm (d, J = 6.4 Hz, 2H), 3.31 ppm (s, 4H), 2.27 ppm (d, J = 2.6 Hz, 6H), 1.19 ppm (s, 8H).

### Microbiology, MIC and MBC Assays

A panel of bacterial strains was selected for antimicrobial testing. Gram-positive strains included *Staphylococcus aureus* (MSSA; ATCC 9144, MRSA; NCTC 13616, USA300, SA-1199b), *Enterococcus faecalis* (VSE; NCTC 775, VRE; NCTC 12201) and *Enterococcus faecium* (VRE; NCTC 12204), while Gram-negative strains included *Klebsiella pneumoniae* (NCTC 13368, M6), *Acinetobacter baumannii* (ATCC 17978, AYE), *Pseudomonas aeruginosa* (PAO1, NCTC 13437), and *Escherichia coli* (NCTC 12923). Additional *K. pneumoniae* and *P. aeruginosa* wild-type and CHX-resistant variants harboring smvR, phoQ, and pmrB mutations, generated previously under serial passage with CHX,^23^ were also used. MICs were determined using the broth microdilution method in 96-well plates following EUCAST guidelines, with concentrations ranging from 0.5 to 512 μg/mL. For MBC determination, 10 μL aliquots were taken from wells showing no visible bacterial growth in the MIC assay and spotted onto TSA agar plates under sterile conditions in a safety cabinet. Plates were incubated overnight at 37 °C and subsequently examined for bacterial regrowth. The lowest concentration of compound that completely prevented bacterial regrowth on agar was recorded as the MBC.

### *Galleria mellonella* Toxicity Assay

The *G. mellonella* larvae were injected with antimicrobial agent/10% DMSO in PBS in the first right proleg. Controls were injected with PBS alone. Ten larvae were treated per dose, per repeat for a total of 30 larvae per dose across 3 independent repeats. *G. mellonella* were stored at 4 °C, allowed to come to room temperature for at least an hour before the procedure and were used within 2 weeks of the receipt date. *G. mellonella* were incubated at 37 °C and assessed for survival every day for 5 days.

### Homology Modeling and Molecular Docking

The homology model of the SmvA efflux pump in *Klebsiella pneumoniae* was developed using AlphaFold 3 with the UniProt sequence A0A378C1V1. Blind docking was first performed using AutoDock SMINA, which applies the AutoDock Vina scoring function to explore potential binding sites across the entire protein structure. Default settings were used, generating nine ligand conformations. The top binding site identified by SMINA was then used for focused, flexible docking with the GOLD software. The best-docked poses were selected based on GOLD fitness scores, with more negative values indicating better binding. Standard cutoffs were used for hydrogen bonding (2.5 Å) and van der Waals interactions (4.0 Å). To validate the docking procedure, we performed a redocking control by redocking chlorhexidine into the previously reported binding pocket^21^ to ensure reproducibility of the binding pose. In addition, docking scores were evaluated against established score thresholds reported in the literature for comparable systems to provide confidence in pose selection. The 2D interaction maps of the best poses were generated using Discovery Studio Visualizer 2021.

## Results

### Design of Chlorhexidine Analogues

The CHX analogues were designed to systematically investigate how different substituents on the terminal aromatic rings influence antibacterial activity, while retaining the core hexamethylene linker essential for amphipathic character and membrane disruption. The parent compound, chlorhexidine (CHX), contains para-chlorophenyl groups, which served as a reference point for substitution. In the analogues, a range of lipophilic and electron-withdrawing groups, including methyl, fluoro, and ether substituents, were introduced at different positions (ortho, meta, and para) to modulate physicochemical properties such as hydrophobicity, electronic distribution, and steric profile (Figure 1).

**Figure 1:**
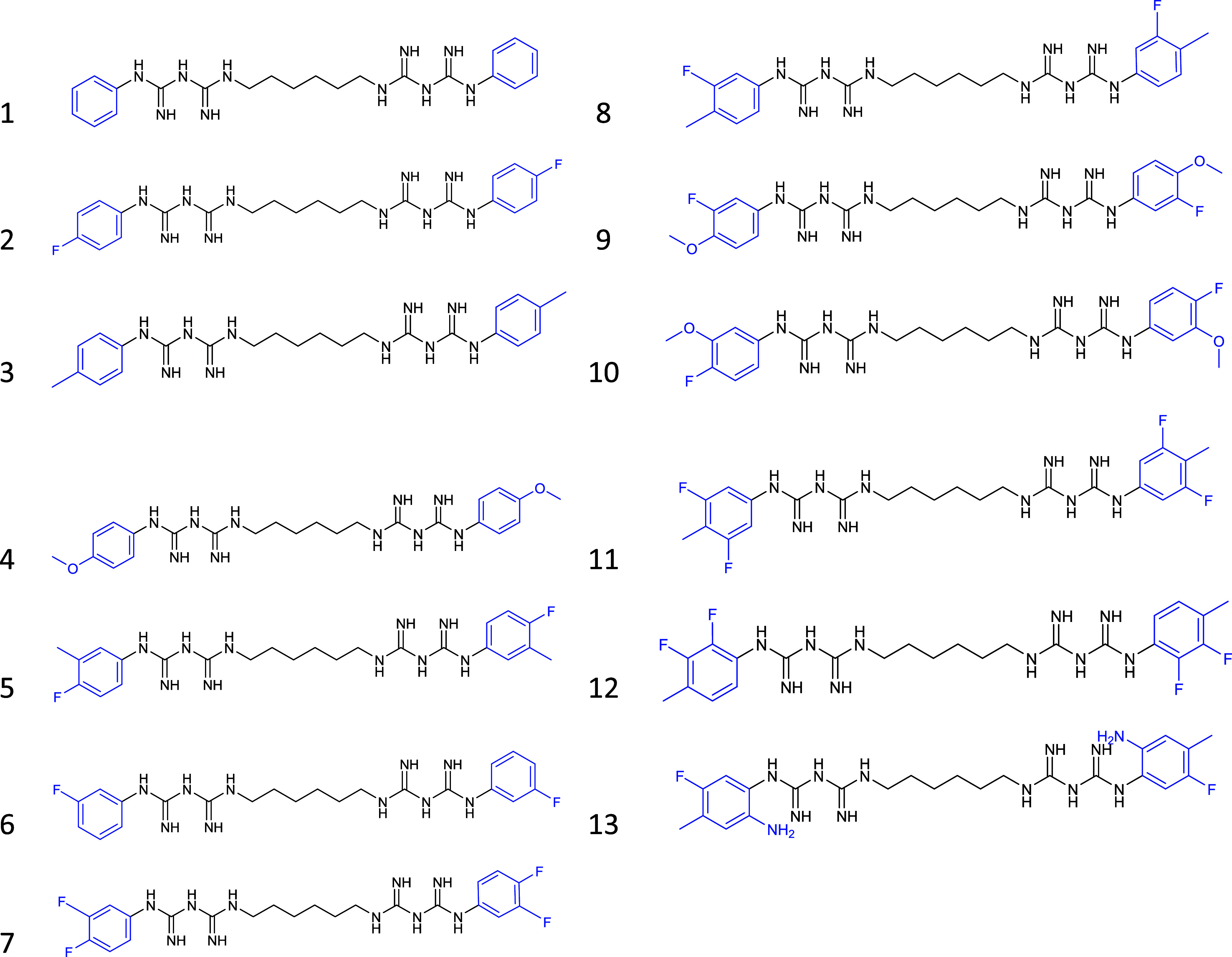
Structures of the chlorhexidine analogues containing the hexamethylene linker and terminal phenyl aromatic rings, in blue, with different substituents.

The design aimed to optimize interactions with bacterial membranes and overcome limitations of CHX, such as reduced efficacy against Gram-negative bacteria and emerging resistance. Specific emphasis was placed on exploring fluorine substitution, given its potential to enhance membrane permeability and reduce efflux susceptibility. Multisubstituted analogues, particularly those combining fluoro and methyl groups at defined positions (e.g., 2-, 3-, or 4-positions), were hypothesized to achieve a balance between potency, spectrum of activity, and resistance evasion.

### Chemical Synthesis

All analogues were synthesized based on the previously described methodology.^36^ The reaction proceeds via nucleophilic attack of the aromatic amine on the terminal cyanoguanidine groups, forming symmetrical bisbiguanide derivatives. The synthesized compounds displayed a wide range of yields and purities, reflecting the influence of electronic and steric properties of the aniline derivatives. Compounds such as **11** had relatively low yields (∼4.3%) but still provided sufficient material for microbiological assays, whereas **5** reached up to 77% yield and high purity. The presence of symmetrical substituents such as 3,5-difluoro and 3,5-dimethyl groups appeared to enhance yield and crystallinity, possibly due to improved intermediate stability during synthesis. The low yields for certain compounds were attributed to incomplete conversion or side reactions, and secondary purification steps via HPLC often led to further loss of material. Nonetheless, final purities exceeded 90% for all compounds, with many achieving >95%, indicating that the compounds were sufficiently pure for biological evaluation.

All 13 analogues were confirmed using LC-MS and HRMS, showing precise alignment with their theoretical molecular weights. HRMS confirmed mass accuracies within <2 ppm, verifying the molecular identities. NMR spectroscopy, particularly HSQC, played a pivotal role in structural validation. For instance, in compound **11**, characteristic proton shifts around 6.9–7.2 ppm corresponded to aromatic protons of the fluorinated aniline moieties, while aliphatic regions (∼1.2–3.1 ppm) confirmed the integrity of the hexamethylene linker. The guanidino groups, essential for CHX-like biocidal activity, were identified through distinct downfield shifts in both proton and carbon spectra. The presence of substituent-specific peaks, such as those associated with −CH_3_ or −OCH_3_ groups, provided further confirmation of the desired structural diversity across the compound library.

### Antibacterial Activity of Chlorhexidine Analogues

#### Microbiological Evaluation of the Compounds

##### Activity against Gram-Positive Bacteria

CHX exhibited potent activity against all Gram-positive strains, with MICs ranging from 0.5 to 4–8 μg/mL (Table 1). The best-performing analogues included compounds **5**, **7**, **8**, and **11**. Compound **11** demonstrated consistent potency across all Gram-positive strains, with MIC values between 1–2 μg/mL, closely matching or improving upon CHX. Compounds **5** and **7** maintained strong activity, showing MICs of 1–16 μg/mL and 2–8 μg/mL, respectively. Compound **8** also showed excellent potency, with MICs ranging from 0.5 to 4 μg/mL. In contrast, several analogues displayed reduced activity. Compounds **1**, **4**, **9** and **10** showed high MIC values against *S. aureus* SA-1199B and the vancomycin-resistant *Enterococcus* (VRE) strains, with MICs ≥ 128 μg/mL in many cases. Compound **2** and **3** showed moderate activity with MICs between 4 and 64 μg/mL.

**Table 1:**
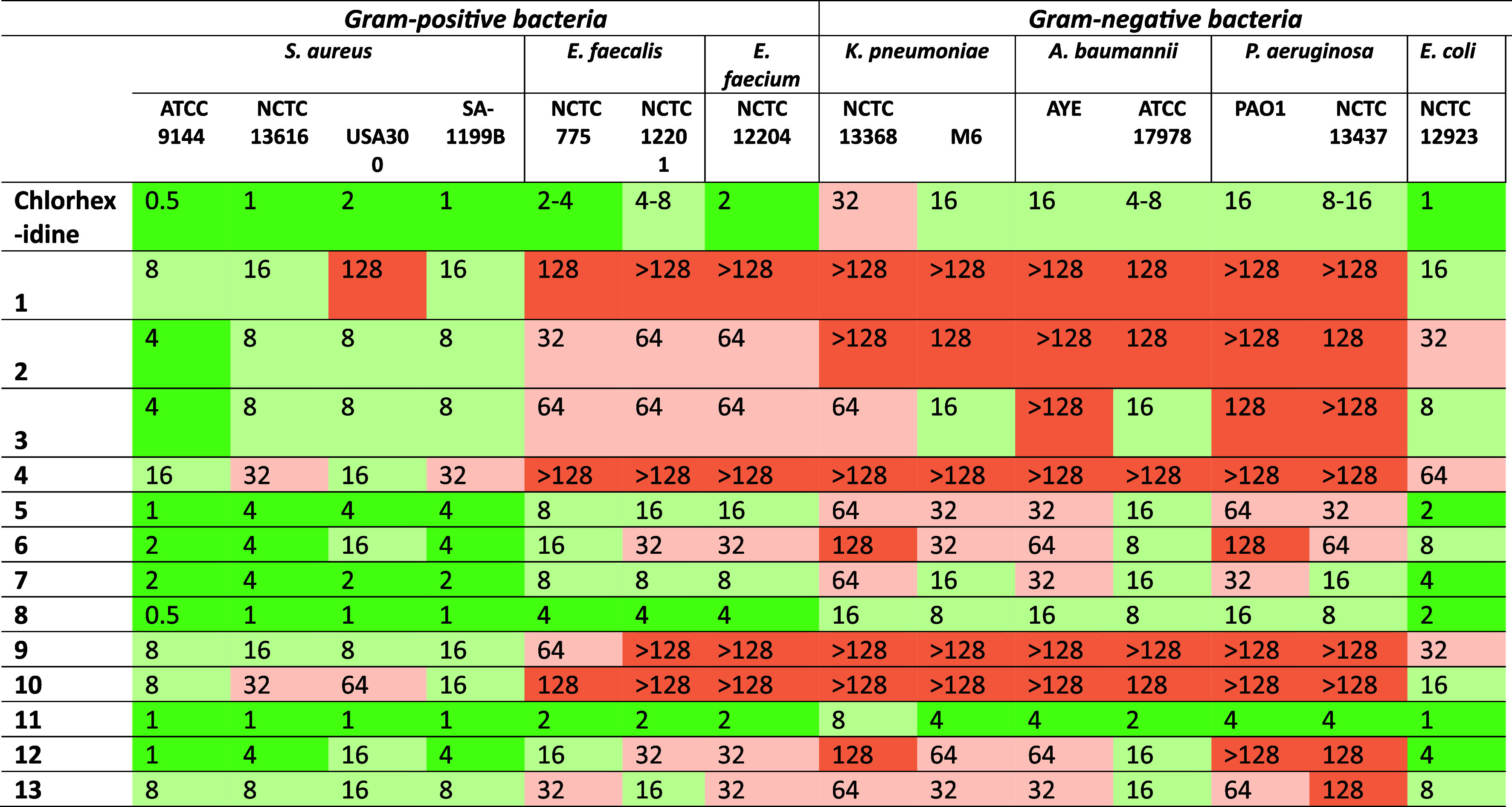
MIC Data of Chlorhexidine Analogues against *Gram-Positive* and *Gram-Negative* Bacterial Strains (μg/mL)

##### Activity against Gram-Negative Bacteria

CHX showed moderate activity against Gram-negative bacteria, with MICs ranging from 1 to 32 μg/mL (Table 1). Compound **11** again demonstrated the most consistent and favorable activity profile among the analogues, with MIC values of 1–8 μg/mL across the tested Gram-negative panel. Compound **8** retained strong activity, particularly against *K. pneumoniae* and *E. coli*, with MICs of 2 to 16 μg/mL. Compounds **5** and **7** also showed moderate to good activity across most strains, with MICs ranging from 2 to 64 μg/mL. Compounds **1**, **2**, **4**, **9**, **10** and **12** exhibited significantly reduced activity against Gram-negative bacteria, especially against multidrug-resistant strains such as *K. pneumoniae* NCTC 13368 and *P. aeruginosa* NCTC 13437, with MICs exceeding 128 μg/mL in most cases. Compound **6** showed variable activity, with MICs ranging from 8 to 128 μg/mL.

##### Activity against Chlorhexidine-Resistant Gram-Negative Bacteria

Based on their promising profiles, compounds **5**, **7**, **8** and **11** were evaluated against a panel of chlorhexidine-resistant *K. pneumoniae* and *P. aeruginosa* strains harboring mutations including *smvR*, *phoQ*, *pmrB*, *fleQ* and *ampDH2*. The MIC values of these compounds were compared with those of chlorhexidine across both wild-type and mutant strains (Table 2).

**Table 2:**
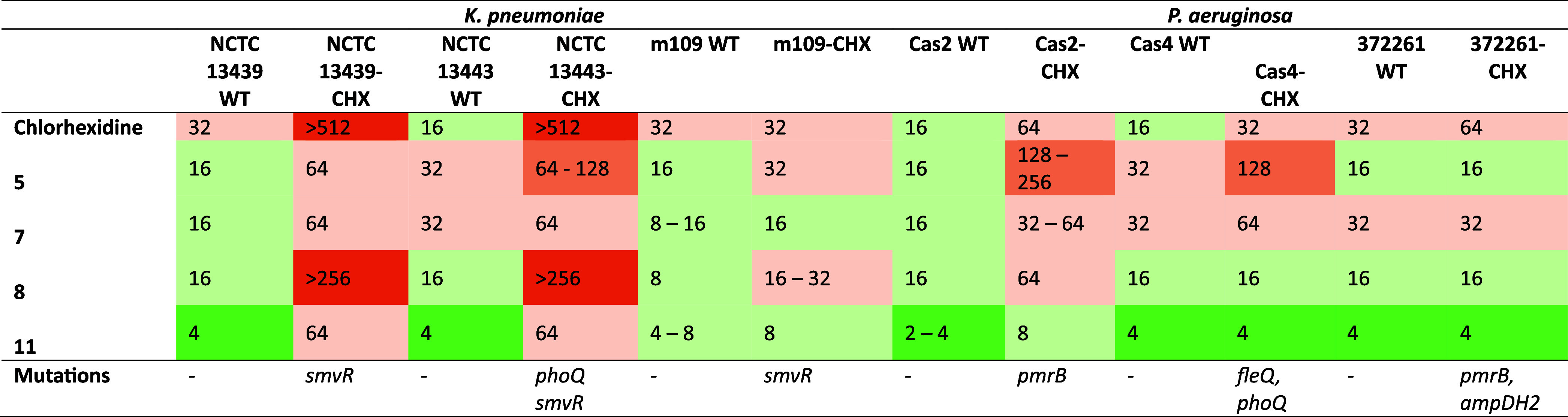
MIC Data of Chlorhexidine Analogues against Wild-Type and CHX-Resistant Strains (μg/mL)

In wild-type strains, **chlorhexidine** showed MIC values of 16–32 μg/mL. In resistant strains, MIC values increased substantially, exceeding 512 μg/mL in *K. pneumoniae* NCTC 13439-CHX and NCTC 13443-CHX, and increasing to 32–64 μg/mL in resistant *P. aeruginosa* strains. In contrast, compound **11** showed the lowest MIC values across the panel, with MICs of 2–8 μg/mL in wild-type strains and retained activity in resistant isolates, with MICs of 64 μg/mL in *K. pneumoniae* NCTC 13439-CHX and NCTC 13443-CHX, 8 μg/mL in *P. aeruginosa* m109-CHX and Cas2-CHX, and 4 μg/mL in Cas4-CHX and 372261-CHX.

Compound **8** showed MIC values of 8–16 μg/mL across wild-type strains, but increased MICs of >256 μg/mL in *K. pneumoniae* resistant strains. In *P. aeruginosa*, compound **8** retained MIC values of 16–64 μg/mL in resistant isolates, including 16–32 μg/mL in m109-CHX, 64 μg/mL in Cas2-CHX, and 16 μg/mL in Cas4-CHX and 372261-CHX. Compound **7** showed MIC values of 8–32 μg/mL in wild-type strains and 16–64 μg/mL in resistant isolates, including 64 μg/mL in both *K. pneumoniae* resistant strains, 16 μg/mL in m109-CHX, 32–64 μg/mL in Cas2-CHX, 64 μg/mL in Cas4-CHX, and 32 μg/mL in 372261-CHX. Compound **5** showed MIC values of 16–32 μg/mL in wild-type strains and 16–256 μg/mL in resistant isolates, including 64 μg/mL in *K. pneumoniae* NCTC 13439-CHX, 64–128 μg/mL in NCTC 13443-CHX, 32 μg/mL in m109-CHX, 128–256 μg/mL in Cas2-CHX, 128 μg/mL in Cas4-CHX, and 16 μg/mL in 372261-CHX.

Minimum bactericidal concentration (MBC) values were determined for the same strain panel (Table 3). In wild-type strains, **chlorhexidine** showed MBC values of 16–64 μg/mL. In resistant strains, MBC values increased markedly, exceeding 512 μg/mL in *K. pneumoniae* NCTC 13439-CHX and NCTC 13443-CHX, and increasing to 64–256 μg/mL across resistant *P. aeruginosa* isolates.

**Table 3:**
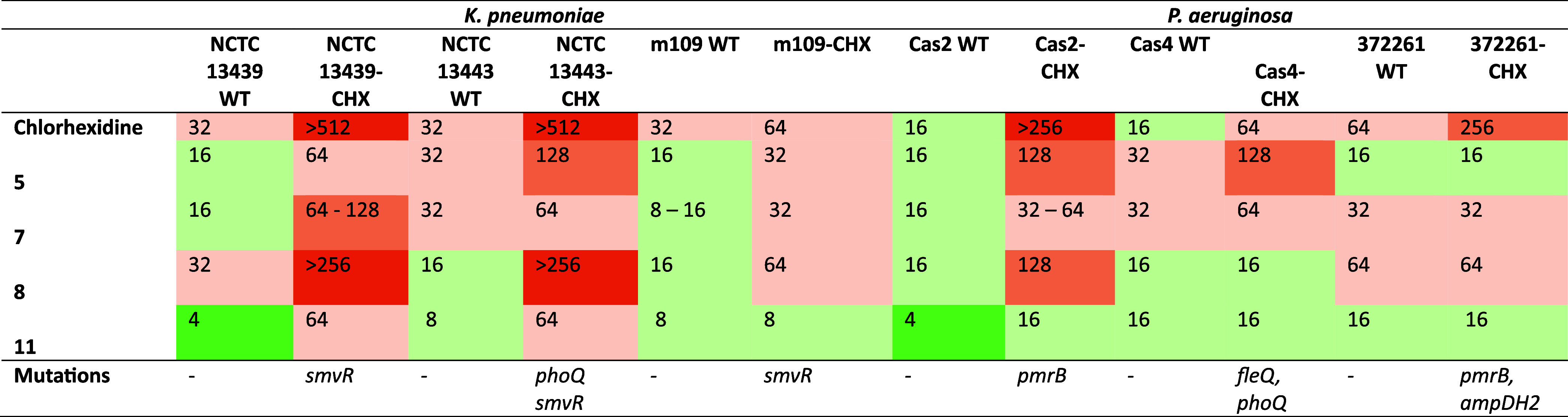
MBC Data of Chlorhexidine Analogues against Wild-Type and CHX-Resistant Strains (μg/mL)

For compounds **5**, **7**, **8** and **11**, MBC values were generally comparable to their corresponding MIC values across both wild-type and resistant strains. Compound **11** showed MBC values of 4–8 μg/mL in wild-type strains and retained activity in resistant isolates, with MBC values of 64 μg/mL in *K. pneumoniae* NCTC 13439-CHX and NCTC 13443-CHX, 8 μg/mL in m109-CHX, and 16 μg/mL in Cas2-CHX, Cas4-CHX and 372261-CHX. Compound **7** showed MBC values of 8–32 μg/mL in wild-type strains and 32–128 μg/mL in resistant isolates, including 64–128 μg/mL in *K. pneumoniae* NCTC 13439-CHX, 64 μg/mL in NCTC 13443-CHX and Cas4-CHX, 32 μg/mL in m109-CHX, 32–64 μg/mL in Cas2-CHX, and 32 μg/mL in 372261-CHX. Compound **5** showed MBC values of 16–32 μg/mL in wild-type strains and 16–128 μg/mL in resistant isolates, including 64 μg/mL in *K. pneumoniae* NCTC 13439-CHX, 128 μg/mL in NCTC 13443-CHX, 32 μg/mL in m109-CHX, and 128 μg/mL in Cas2-CHX and Cas4-CHX. Compound **8** showed MBC values of 16–64 μg/mL in wild-type strains and increased values in resistant isolates, including >256 μg/mL in *K. pneumoniae* NCTC 13439-CHX and NCTC 13443-CHX, 64 μg/mL in m109-CHX, 128 μg/mL in Cas2-CHX, and 64 μg/mL in 372261-CHX.

Across all strains tested, chlorhexidine showed consistently higher MBC values in resistant isolates compared to wild-type strains. In contrast, for the fluorinated analogues, MIC and MBC values were identical or closely aligned in most cases, with increases in MBC relative to MIC observed for specific strain–compound combinations.

### Molecular Modeling

To explore the basis for the reduced efflux liability and improved activity of compound **11** against chlorhexidine-resistant strains, particularly *K. pneumoniae*, we compared its molecular interactions within the SmvA efflux pump binding pocket to those of **chlorhexidine**. This comparison was motivated by our previous findings that chlorhexidine resistance in *K. pneumoniae* is associated with the overexpression of the SmvA efflux pump.^21^ The 2D interaction maps of compound **11** and **chlorhexidine** provide insight into the differential efflux liabilities of these compounds through SmvA. Compound **11** forms an extensive network of favorable interactions with SmvA, including conventional hydrogen bonds with Asn130, Arg120, and Met131, which likely contribute to stabilizing its conformation within the binding pocket (Figure 2). In addition, pi-sulfur interactions with Met377 and Met131, along with pi-alkyl and alkyl interactions involving Pro313, Ala385, and Arg120, suggest a strong hydrophobic contribution to its binding mode. Further stabilization is provided by carbon–hydrogen bonding and a pi–cation interaction with Arg129. The fluorine and methyl substitutions on the terminal phenyl ring appear to play critical roles in facilitating these interactions. This diverse interaction profile, particularly the strong hydrophobic contacts, is likely responsible for the reduced efflux liability of compound **11**. Previous work by our group and others has demonstrated that such hydrophobic interactions with efflux pump residues can modulate pump dynamics and reduce the likelihood of compound extrusion. In contrast, the interaction profile of **chlorhexidine** (Panel B) appears less extensive and structurally less favorable. Although it forms hydrogen bonds with Asn130, it also exhibits an unfavorable positive–positive electrostatic interaction with Arg129. Its hydrophobic contacts, including alkyl and van der Waals interactions with Ala126, Leu317, and Met131, are comparatively limited and do not provide the same degree of stabilization observed for compound **11**.

**Figure 2:**
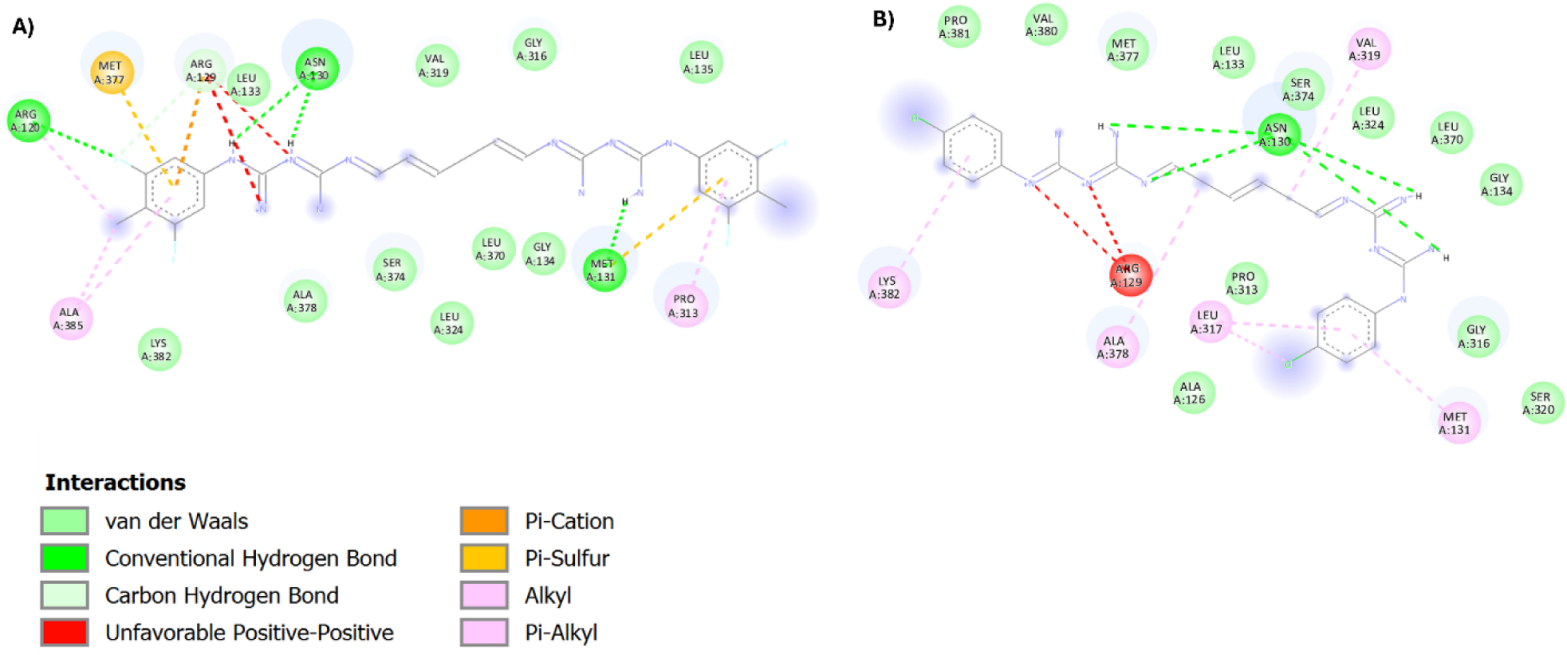
2D interaction map of compound **11** (A) and **chlorhexidine** (B) within the binding pocket of SmvA in *Klebsiella pneumoniae*.

### Toxicity Study

The nonselective toxicity of the compounds was assessed using a *Galleria mellonella* toxicity assay, a well-established nonmammalian model for evaluating compound toxicity. Compounds **8** and **11**, two of the most active candidates, were selected for this assay alongside **chlorhexidine**. The survival plot (Figure 3) demonstrates that Compounds **8** and **11** exhibit minimal toxicity across all tested doses, with survival rates remaining consistently high over the full 120-h observation period. For Compound **8**, larvae treated at 10, 20, and even 50 mg/kg showed no significant mortality, indicating strong tolerability and low systemic toxicity. Similarly, Compound **11** maintained high survival across all doses, with only a modest reduction at 50 mg/kg and survival still exceeding 90%. In contrast, **chlorhexidine** showed a clear dose-dependent decline in survival, with mortality evident even at 20 mg/kg and increasing to approximately 20% at 50 mg/kg. These findings suggest that Compounds **8** and **11** are better tolerated, or at least exhibit a comparable toxicity profile to **chlorhexidine**, highlighting their superior safety and potential suitability for higher-exposure applications.

**Figure 3:**
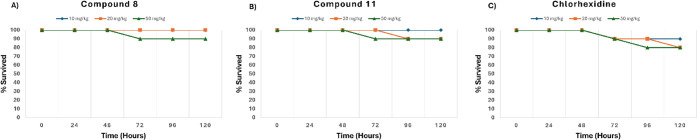
*Galleria mellonella* toxicity data for compounds **8** (A) and **11** (B) show comparable or superior toxicity profile compared to **chlorhexidine** (C).

## Discussion

The chlorhexidine (CHX) analogues synthesized in this study were designed to explore the impact of various substituents on the terminal aromatic rings while retaining the core hexamethylene linker. The parent compound, CHX, contains two para-chlorophenyl moieties, which contribute to its amphipathic structure and membrane-disrupting properties. Structural modifications introduced across compounds **1**–**13** included methyl, fluoro, and ether substituents at various positions on the phenyl ring to systematically assess structure–activity relationships.

Overall, several trends emerged from the antimicrobial activity profiles against the standard panel of Gram-positive and Gram-negative bacteria. Compounds **5**, **7**, **8** and **11** were consistently more potent than other analogues and displayed MIC values that were comparable to, or better than, CHX. Compound **11**, in particular, showed strong and uniform activity across both Gram-positive and Gram-negative strains of the standard panel. Its superior potency may be attributed to a combination of electron-withdrawing and lipophilic substitutions, which could enhance membrane interaction and bacterial uptake.

Compound **5**, which contained a para-fluoro and meta-methyl substitution on the aromatic rings, maintained good activity across a broad range of bacteria. Compound **7**, featuring difluoro substitution at the para- and meta-positions of the phenyl ring, also retained low MICs. Compound **8**, containing para-methyl and meta-fluoro substituents, showed good activity against both Gram-positive and Gram-negative bacteria of the standard panel. In contrast, analogues without any substitutions or those with polar substituents (e.g., compounds **1**, **4**, **9** and **10**) showed markedly diminished activity.

Among Gram-positive bacteria, nearly all active analogues containing methyl and fluoro substitutions at different positions showed good MICs, suggesting that the position of fluorine or methyl groups on the terminal phenyl ring did not significantly impair activity in organisms with relatively permeable membranes. However, compound **1**, without any substitution, showed weak activity against Gram-positive bacteria, and monosubstituted compounds **2**, **3**, **4** and **6** showed significantly less activity compared to di- or trisubstituted compounds, particularly where the substituents were methyl or fluorine. The activity of the compounds against Gram-negative strains varied more widely, likely due to the presence of outer membrane barriers and efflux systems. Notably, CHX showed diminished activity against certain Gram-negative isolates such as *K. pneumoniae* NCTC 13368 and *P. aeruginosa* NCTC 13437, and among the synthesized compounds, only compounds **8** and **11** showed good activity against these strains.

In the extended panel comprising CHX-resistant *K. pneumoniae* and *P. aeruginosa* strains, compounds **8** and **11** showed superior activity compared to CHX, while compounds **5** and **7** showed comparable activity against most strains and better activity against some strains. These strains contained well-characterized resistance mutations, including *smvR*, *phoQ*, *pmrB*, *fleQ*, and *ampDH2*, which are associated with increased efflux activity, membrane modification, and altered surface charge mechanisms known to reduce susceptibility to cationic biocides such as CHX.

CHX was either inactive or only moderately active against these CHX-resistant strains, with MICs often exceeding 512 μg/mL. Compound **8**, bearing a para-methyl and a meta-fluoro substitution, showed better activity than CHX against CHX-resistant *P. aeruginosa* strains but was not able to overcome resistance in CHX-resistant *K. pneumoniae* strains. Compound **5**, with a fluoro group in the para-position and a methyl group in the meta-position, and the structurally related difluoro-substituted compound **7**, failed to overcome resistance in CHX-resistant *P. aeruginosa* strains but showed significantly better activity against CHX-resistant *K. pneumoniae* strains NCTC 13439-CHX and NCTC 13443-CHX.

In contrast, the trisubstituted compound **11**, featuring a para-methyl group combined with a 3,5-difluoro pattern, showed lower MIC values (4–16 μg/mL) across the CHX-resistant strains. This indicates that the specific spatial arrangement of the para-methyl and the two fluorine substituents, rather than the degree of substitution alone, may influence key physicochemical properties—such as electron distribution, local hydrophobicity, and ionizable environment—and thereby affect how the compound interacts with the efflux pump. The molecular modeling study revealed that compound **11** formed a broad and favorable interaction network with the SmvA efflux pump, including hydrogen bonds and hydrophobic contacts with key residues such as Asn130, Arg120, and Met131. In contrast, **chlorhexidine** showed fewer stabilizing interactions and even unfavorable electrostatic contacts, which may contribute to its poorer activity against efflux-driven resistance.

The MBC data generated in this study further support these observations. Chlorhexidine showed consistently higher MBC values in CHX-resistant strains compared to wild-type strains, consistent with reduced bactericidal activity in resistant backgrounds. In contrast, for compounds **5**, **7**, **8** and **11**, MBC values were generally comparable to their corresponding MIC values across both wild-type and resistant strains, which is consistent with the known biocidal mode of action of this class. Notably, compound **11** maintained low MBC values across both wild-type and resistant *P. aeruginosa* strains, with increases primarily observed in CHX-resistant *K. pneumoniae* strains. Compounds **5** and **7** also showed broadly similar MIC and MBC values across many strains, although increases in MBC were observed in specific resistant isolates, particularly in *K. pneumoniae*. In contrast, compound **8** showed greater divergence between MIC and MBC values in several resistant strains, particularly in *K. pneumoniae* and selected *P. aeruginosa* isolates.

These observations suggest that modifications to the terminal phenyl ring, particularly substitution patterns that combine lipophilicity with steric or electronic features, can help bypass resistance mechanisms associated with CHX. The number and combination of fluoro and small alkyl substitutions also appear to be important, as di- and trisubstituted analogues were generally more active than monosubstituted compounds, except when one of the substitutions introduces significant hydrophilicity. The para-chloro groups in CHX may be more readily recognized or expelled by bacterial efflux pumps, or may be less efficient at disrupting altered membranes in resistant strains.

Taken together, these results highlight the value of rational design in tuning the physicochemical properties of CHX analogues to improve both spectrum and potency, particularly in the context of emerging resistance.

## Conclusion

This study demonstrates the successful design of chlorhexidine (CHX) analogues with improved antibacterial activity through strategic modification of the terminal aromatic rings. By introducing fluoro and methyl substituents at specific positions, several analogues, particularly compounds **5**, **7**, **8** and **11**, demonstrated enhanced potency against both wild-type and CHX-resistant Gram-positive and Gram-negative bacteria. These compounds generally showed comparable MIC and MBC values across the strain panel, consistent with a biocidal mode of action, with some divergence observed in specific resistant isolates. Compound **11**, featuring a para-methyl and 3,5-difluoro substitution, consistently showed the lowest MICs and retained low MBC values across resistant strains. These findings highlight the importance of combining lipophilic and electron-withdrawing groups to overcome resistance mechanisms. Overall, the study provides a clear structure–activity framework for guiding the development of next-generation CHX-based biocides.

## Supplementary Material



## Data Availability

All data supporting the findings of this study are included in the main manuscript and the Supporting Information. Additional raw data or materials can be made available by the corresponding author upon reasonable request.

## Notes

LC-MS method; NMR spectra; HRMS (PDF)
