# Supplementary material for: Novel Fluorinated Chlorhexidine Analogues Overcome Resistance in Gram-Negative Bacteria
Source: ACS Omega. 2026 Apr 28;11(18):26382–92. doi: 10.1021/acsomega.5c11738 (PMC13177218; doi:10.1021/acsomega.5c11738)
Supplement: Supplementary file 1 [file ao5c11738_si_001.pdf]

Novel Fluorinated Chlorhexidine Analogues Overcome Resistance in Gram-negative Bacteria

Yunxiao Li[1], Maida Jajja[1], Jiajing Lu[1], Nasima Chowdhury[1], Charlotte Hind[2], J. Mark Sutton[2] and Khondaker Miraz Rahman*[1]

[1]*School of Cancer and Pharmaceutical Sciences, King's College London, Franklin-Wilkins Building, 150 Stamford Street, London SE1 9NH, United Kingdom.*

[2] *Countermeasures Development, Evaluation and Preparedness, Public Health Microbiology, UK Health Security Agency, Manor Farm Road, Porton Down, Salisbury SP4 0JG, United Kingdom.*

Corresponding author. Tel: +44-(0)771-731-2299; E-mail: k.miraz.rahman@kcl.ac.uk

Supporting Information

Purity determination of synthesized final compounds

The level of purity of the compounds for biological testing has been evaluated through LC-MS analysis, using two different gradient methods, reported hereafter. LC-MS analyses were performed on a Waters Alliance 2695 system (from Waters), with elution in gradient. HPLC grade solvents were used as mobile phase while a Monolithic C18 50 X 4.60 mm column (from Phenomenex) was used as stationary phase. UV detection was performed using a Waters 2996 photo array detector (from Waters). Injection volume has been set to 10 µL. The compounds have been dissolved in a mixture of H2O/ACN (50/50, v/v) or DMSO/ACN (50/50, v/v) accordingly to the solubility. The area of the peak corresponding to the compound has been automatically determined by the software included in the LC-MS system. The eventual presence of solvent UV trace has been subtracted to the total in order to determine the percentage of purity. All compounds showed at least 95% purity in both methods.

LC-MS methods:

Method A: flow 0.5 mL/min

A) water + 0.1 % formic acid

B) acetonitrile + 0.1% formic acid

Time (min)	0	3	3.5	4.5	5
A (%)	95	10	5	5	95
B (%)	5	90	95	95	5

Method B: flow 1 mL/min

A) water + 0.1 % formic acid

B) acetonitrile + 0.1% formic acid

Time (min)	0	2	5	6	7.5	9	10
A (%)	95	95	50	50	5	95	95
B (%)	5	5	50	50	95	5	5

NMR and MS Spectra of Final Compounds

N-phenyl-1-[N'-(6-{N-[(N'-phenylcarbamimidamido methanimidoyl]amino}hexyl)carbamimidamido]methanimidamide (1)

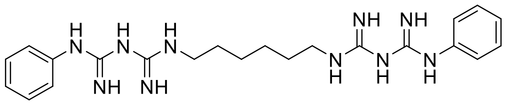

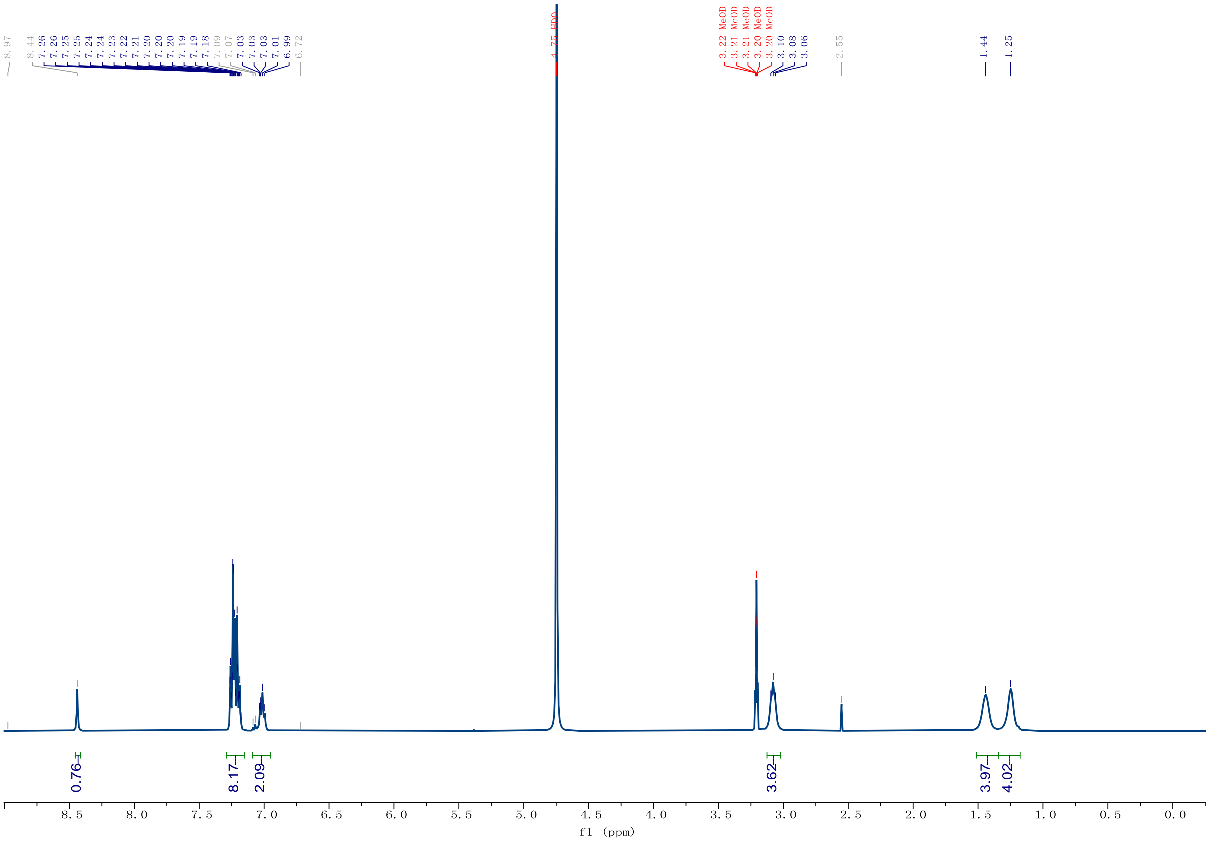

Figure S1-1: Proton ^{1}H NMR spectrum of Compound **1.**

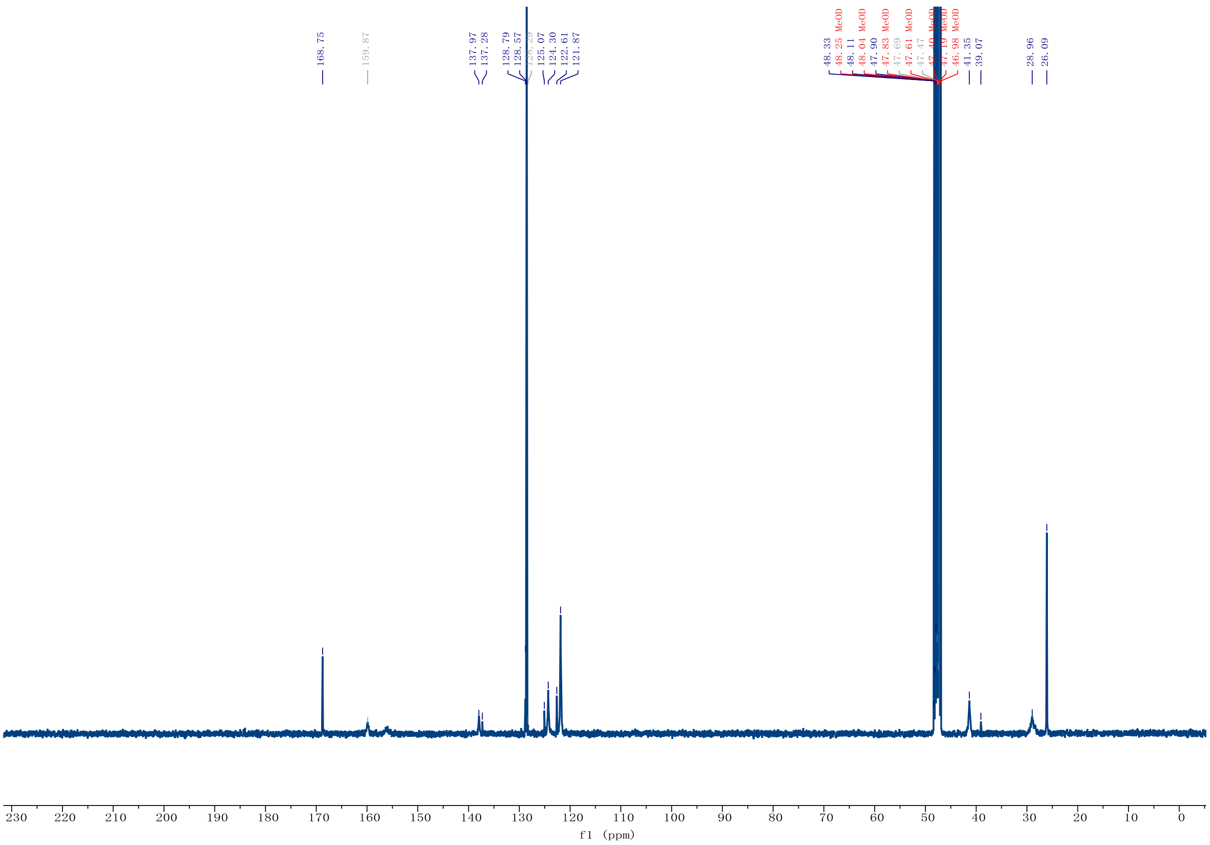

Figure S1-2: Carbon ^{13}C NMR spectrum of Compound **1.**

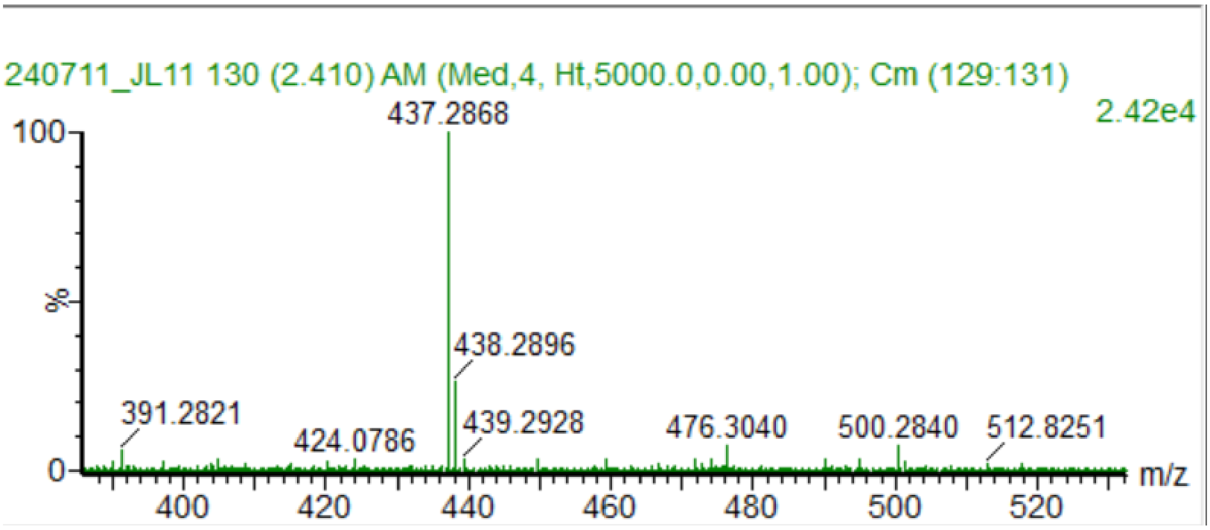

Figure S1-3: HRMS spectrum of Compound **1.**

N-(4-fluorophenyl)-1-{N'-[6-(N-{[N'-(4-fluorophenyl)carbamimidamido]methanimidoyl}amino)hexyl]carbamimidamido}methanimidamide (2)

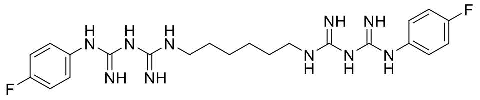

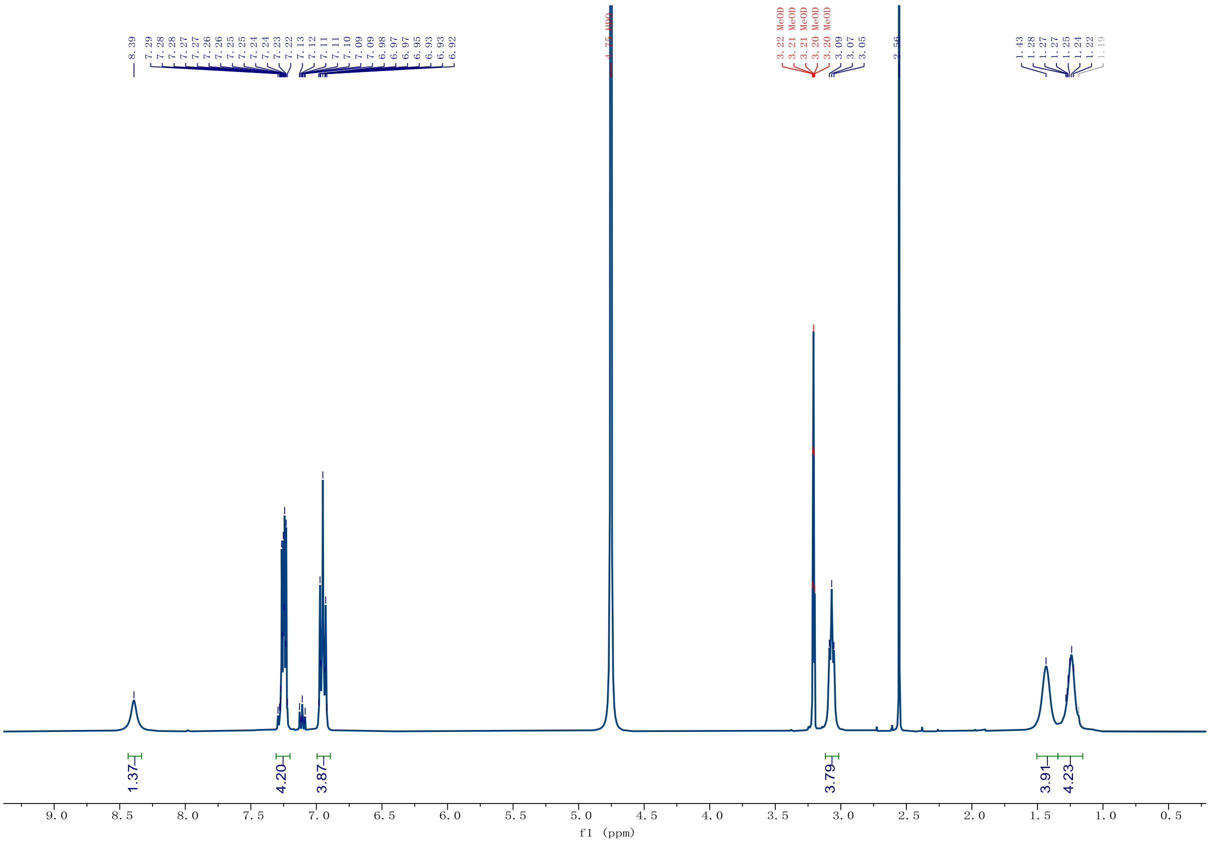

Figure S2-1: Proton ^{1}H NMR spectrum of Compound **2.**

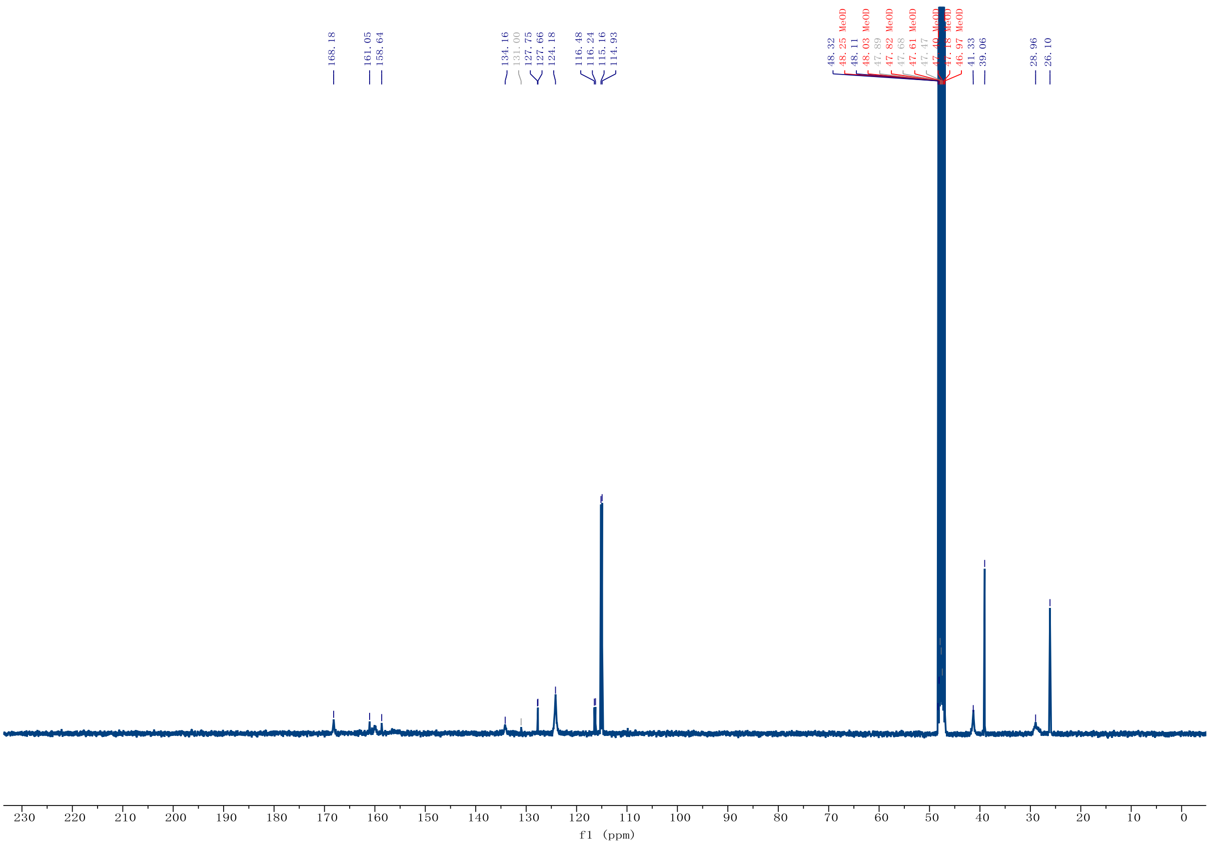

Figure S2-2: Carbon ^{13}C NMR spectrum of Compound **2.**

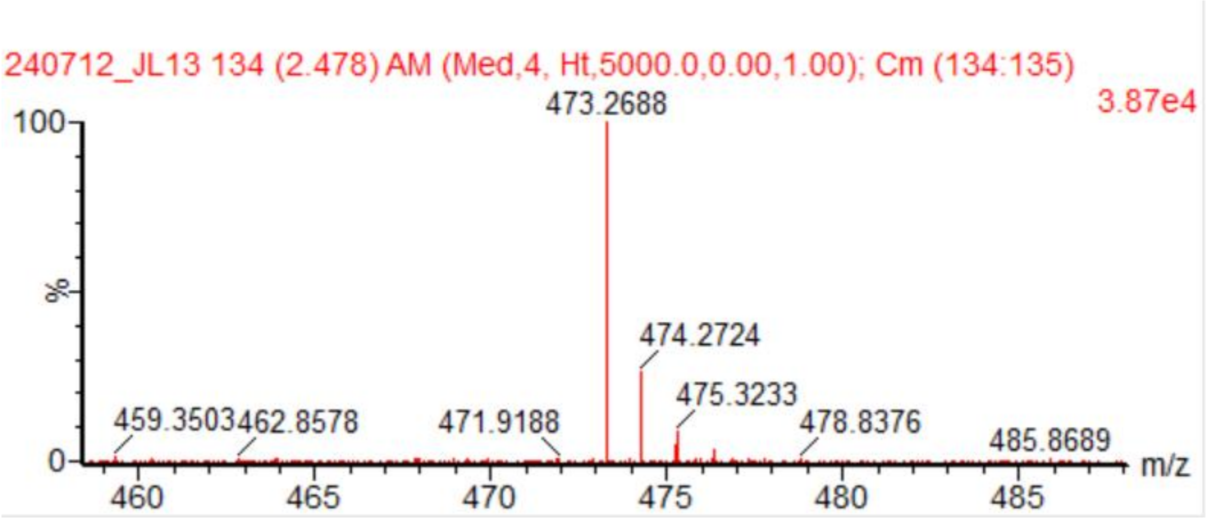

Figure S2-3: HRMS spectrum of Compound **2.**

N-(4-methylphenyl)-1-{N'-[6-(N-{[N'-(4-methylphenyl)carbamimidamido]methanimidoyl}amino)hexyl]carbamimidamido}methanimidamide (3)

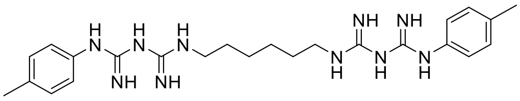

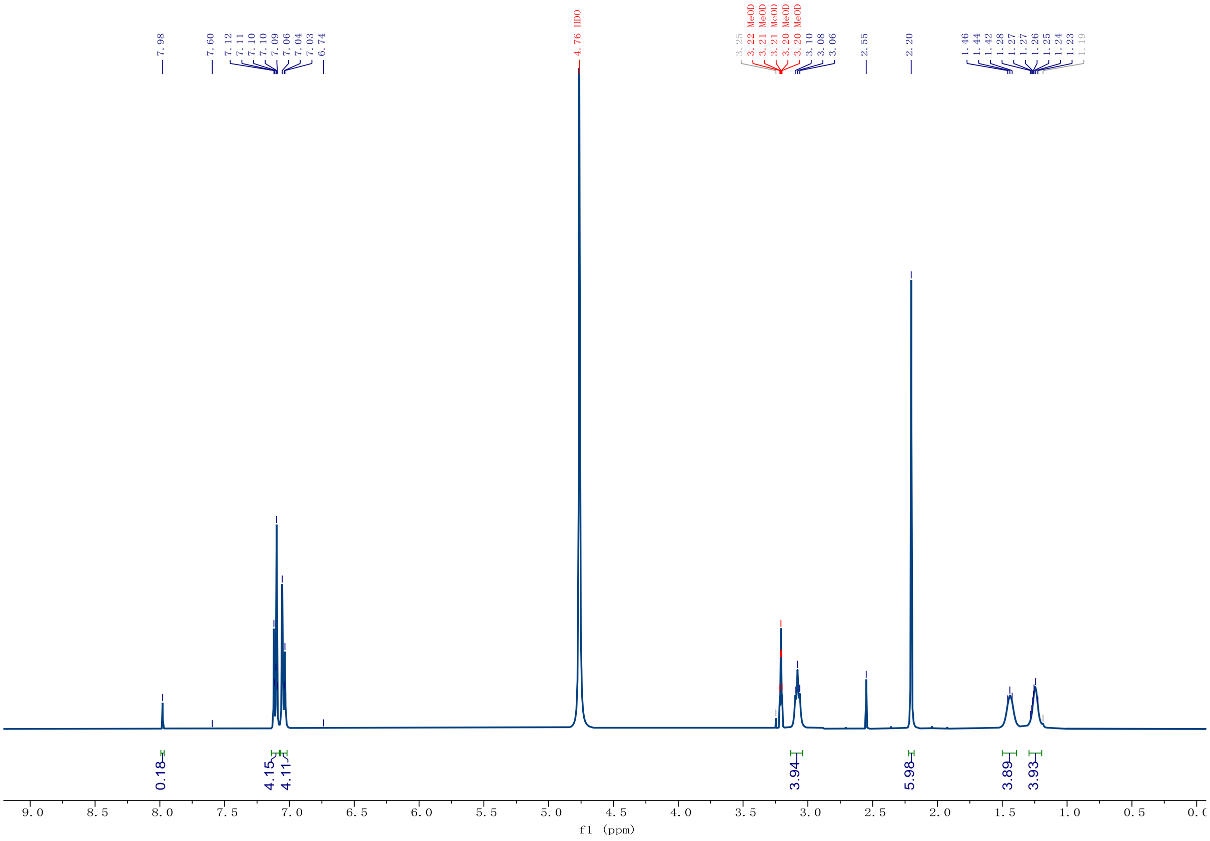

Figure S3-1: Proton ^{1}H NMR spectrum of Compound **3.**

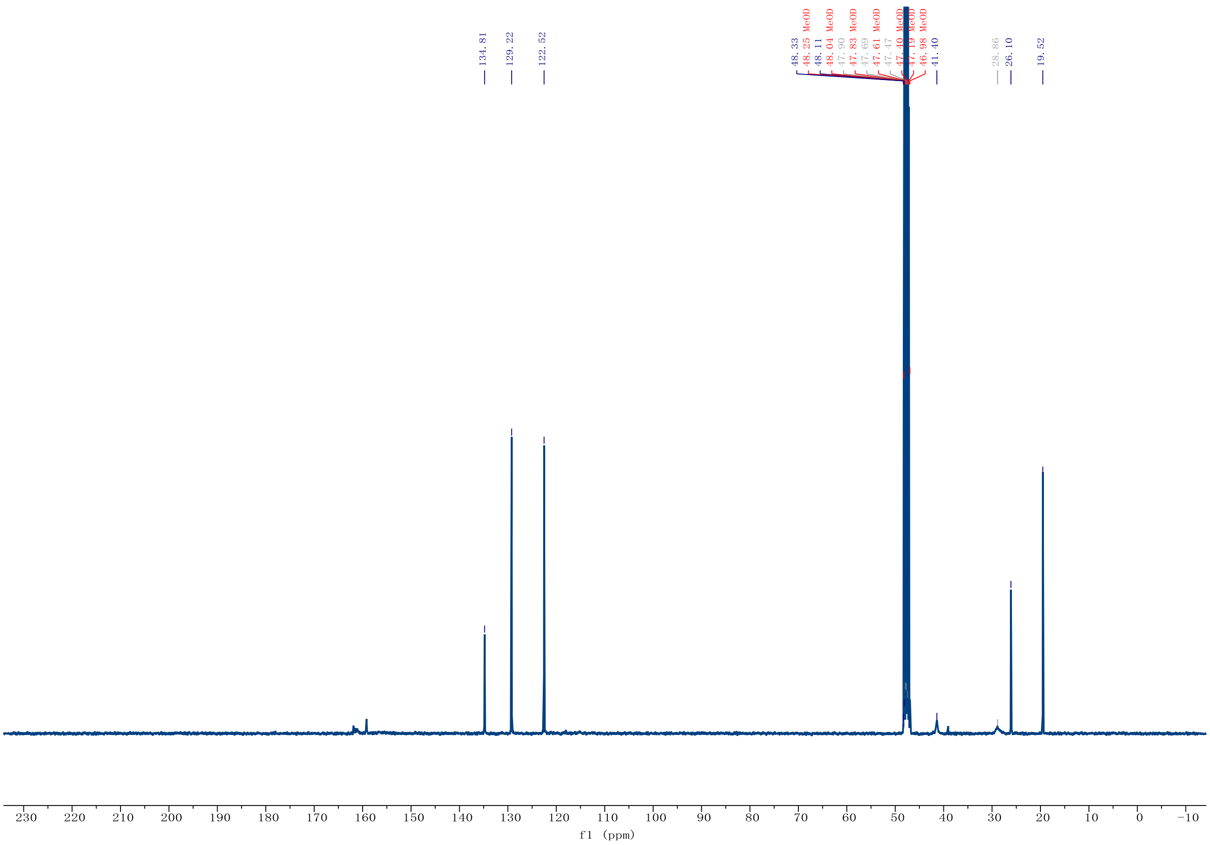

Figure S3-2: Carbon ^{13}C NMR spectrum of Compound **3.**

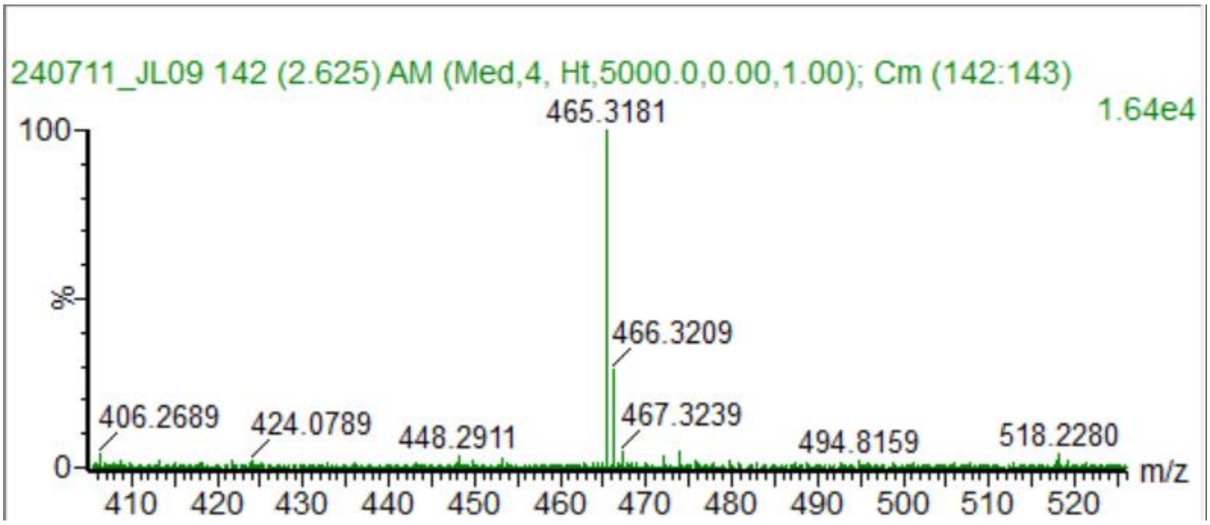

Figure S3-3: HRMS spectrum of Compound **3.**

N-(4-methoxyphenyl)-1-{N'-[6-(N-{[N'-(4-methoxyphenyl)carbamimidamido]methanimidoyl}amino)hexyl]carbamimidamido}methanimidamide (4)

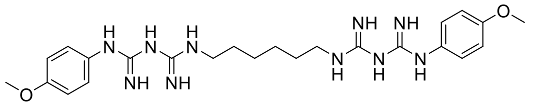

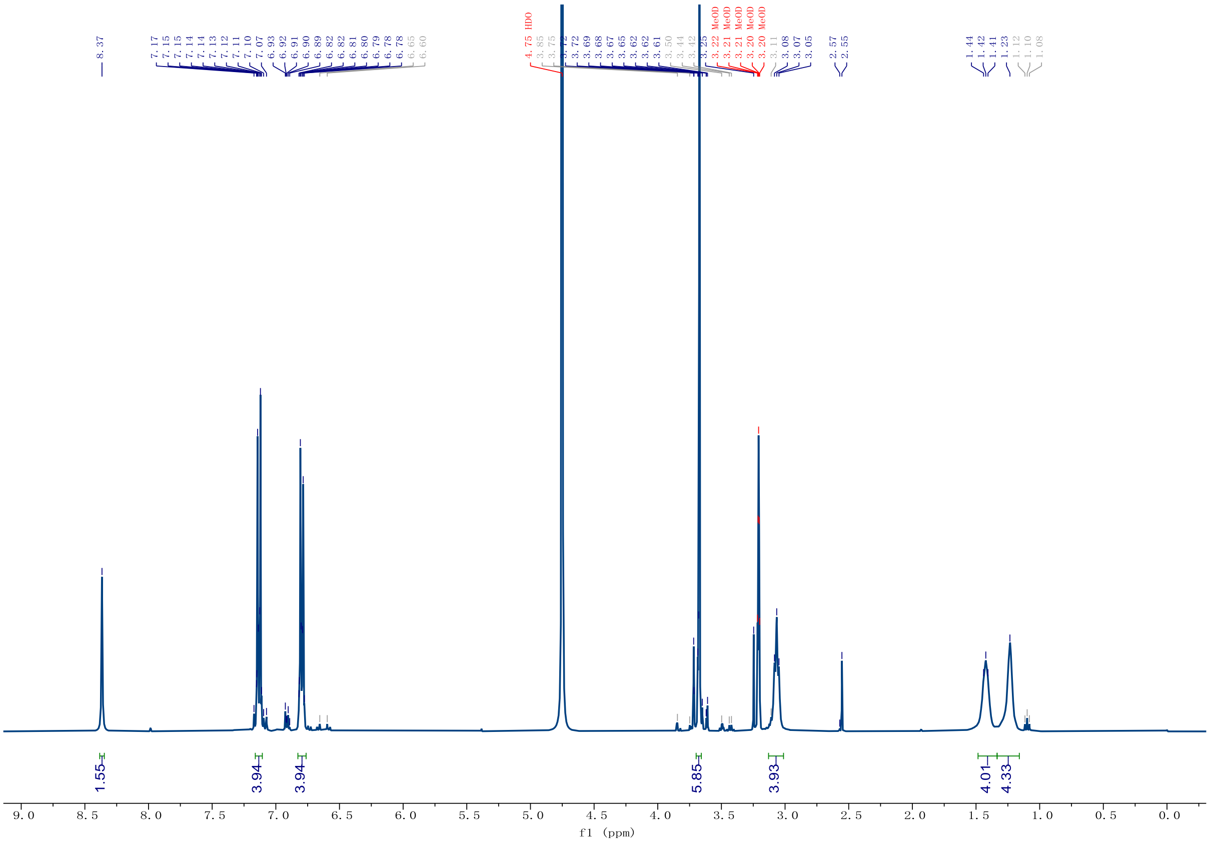

Figure S4-1: Proton ^{1}H NMR spectrum of Compound **4.**

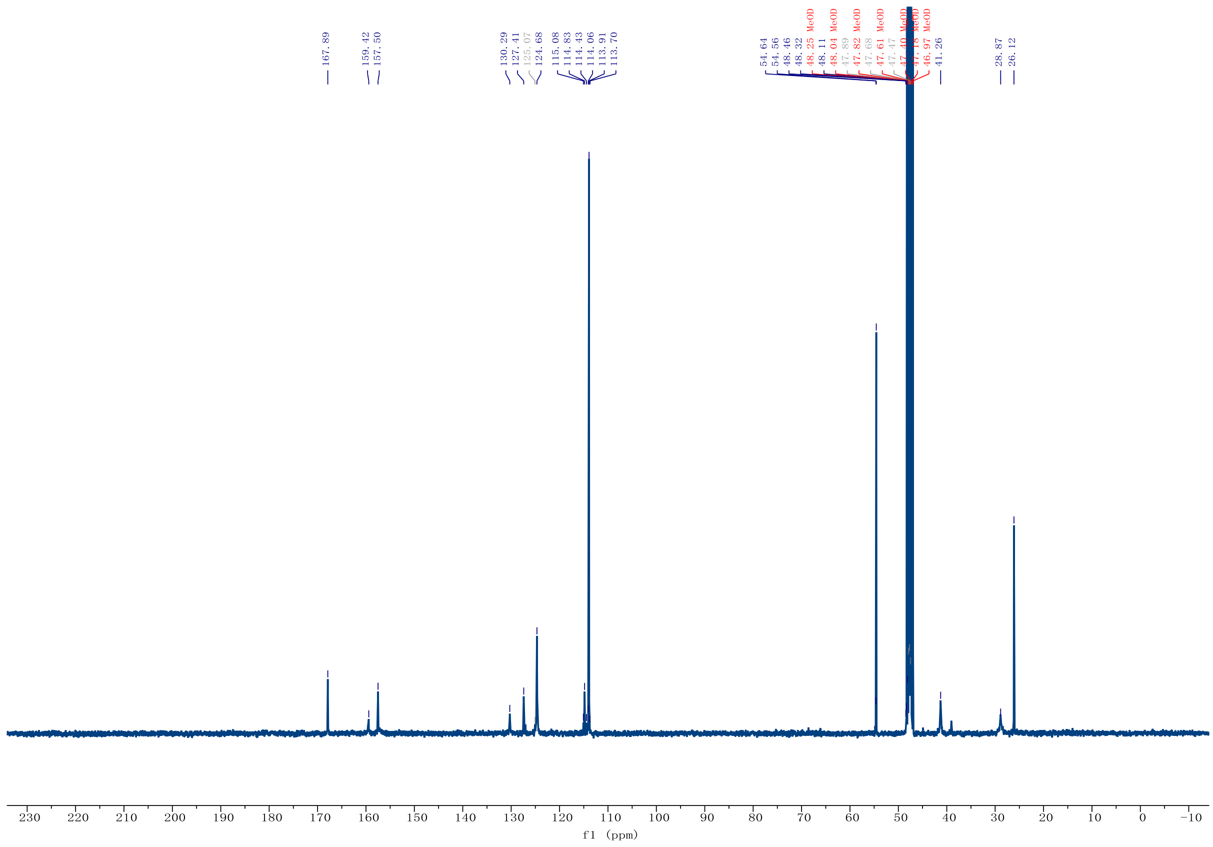

Figure S4-2: Carbon ^{13}C NMR spectrum of Compound **4.**

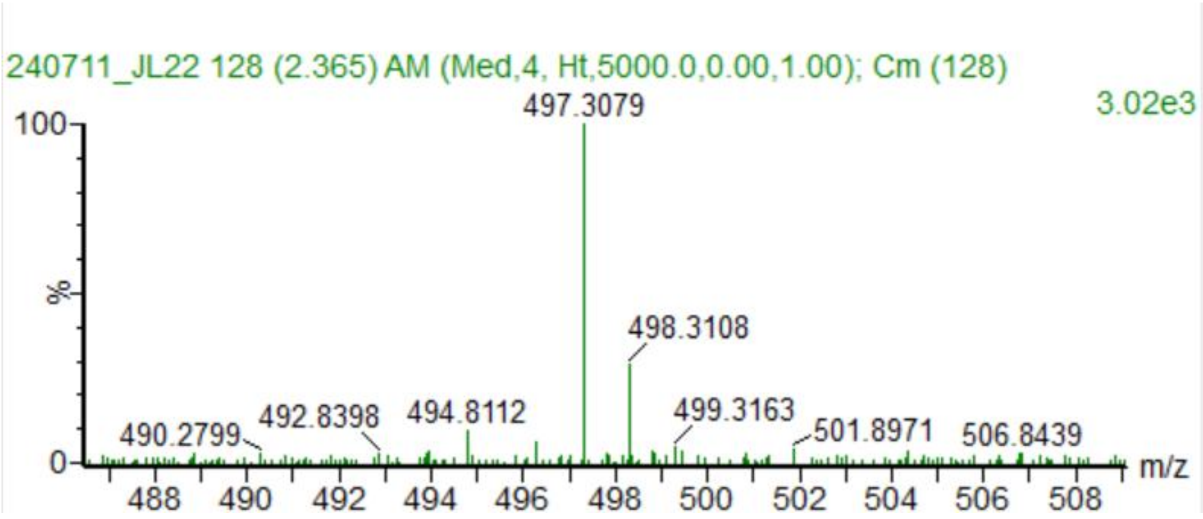

Figure S4-3: HRMS spectrum of Compound **4.**

N-(4-fluoro-3-methylphenyl)-1-{N'-[6-(N-{[N'-(4-fluoro-3-methylphenyl)carbamimidamido]methanimidoyl}amino)hexyl]carbamimidamido}methanimidamide (5)

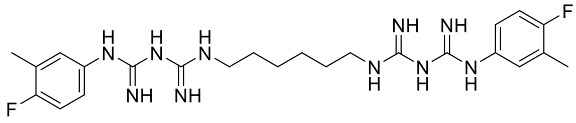

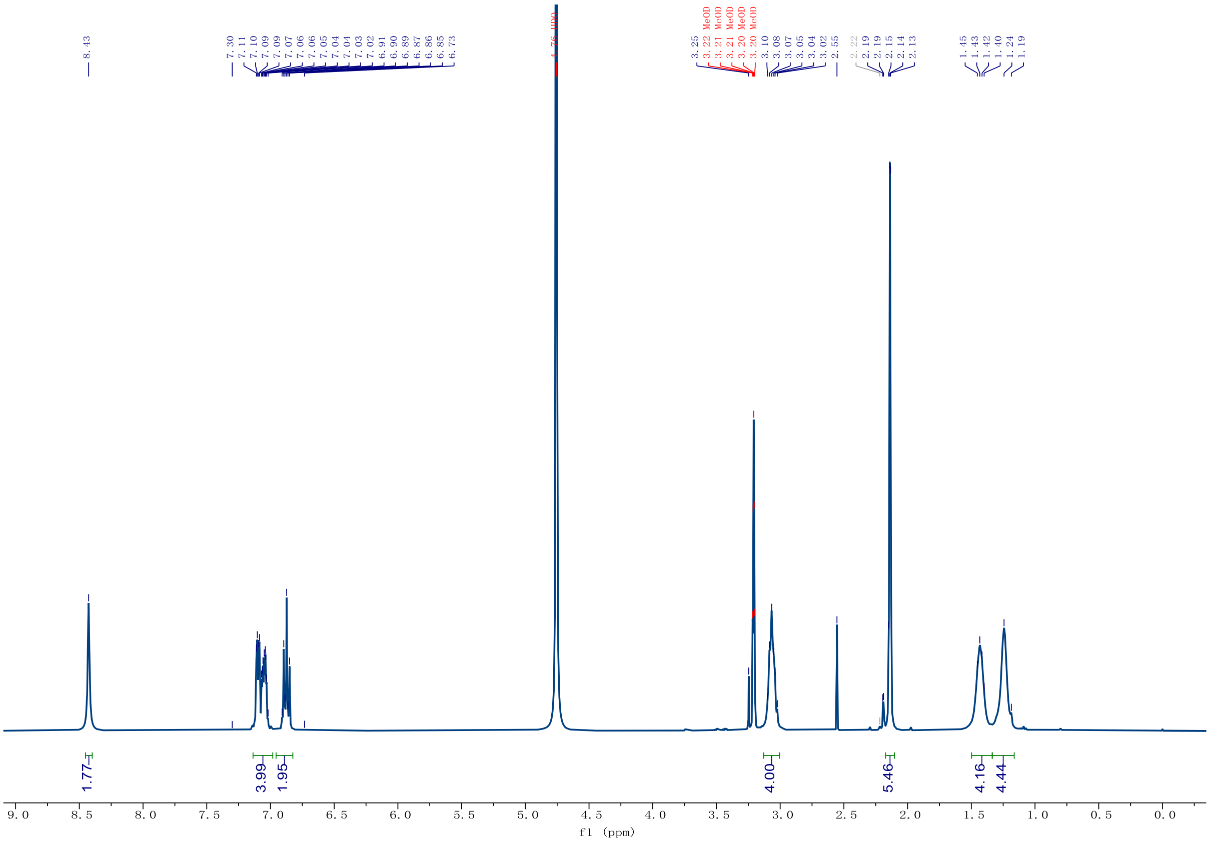

Figure S5-1: Proton ^{1}H NMR spectrum of Compound **5.**

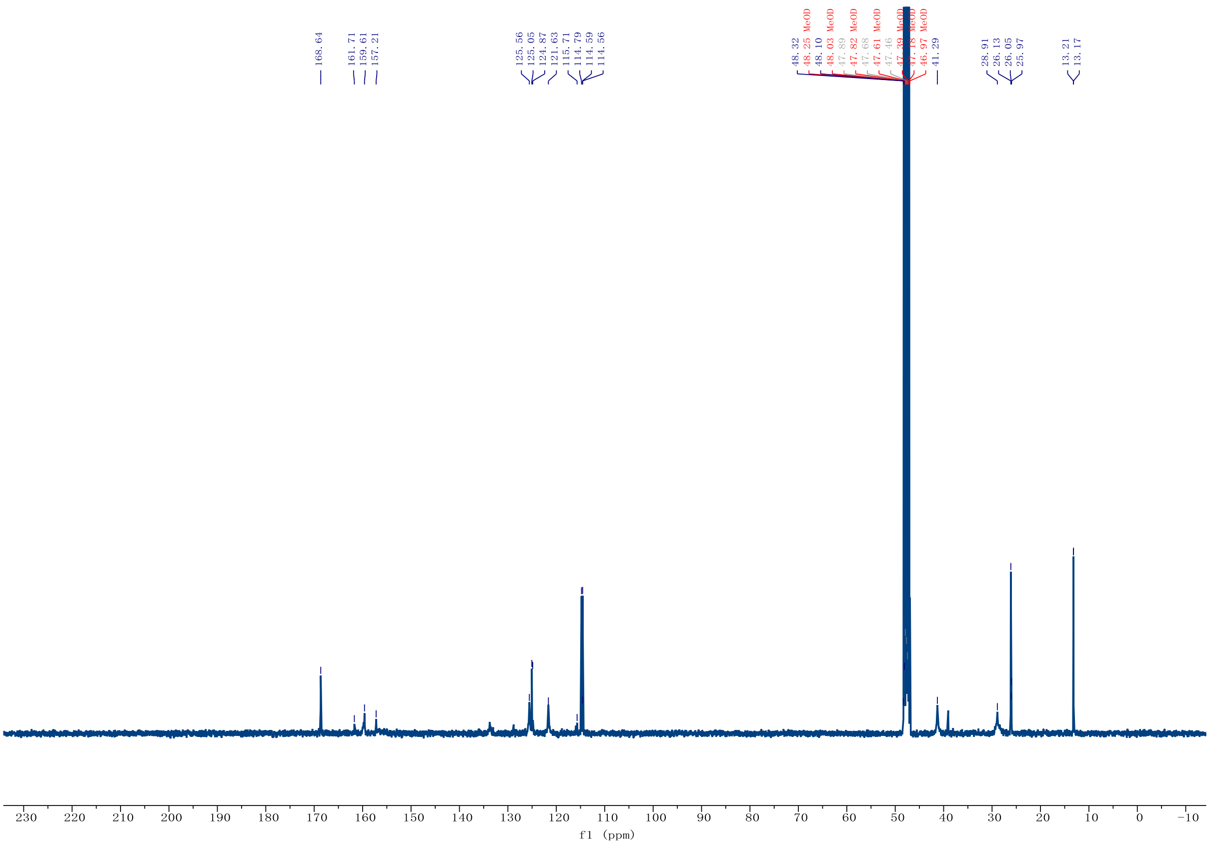

Figure S5-2: Carbon ^{13}C NMR spectrum of Compound **5.**

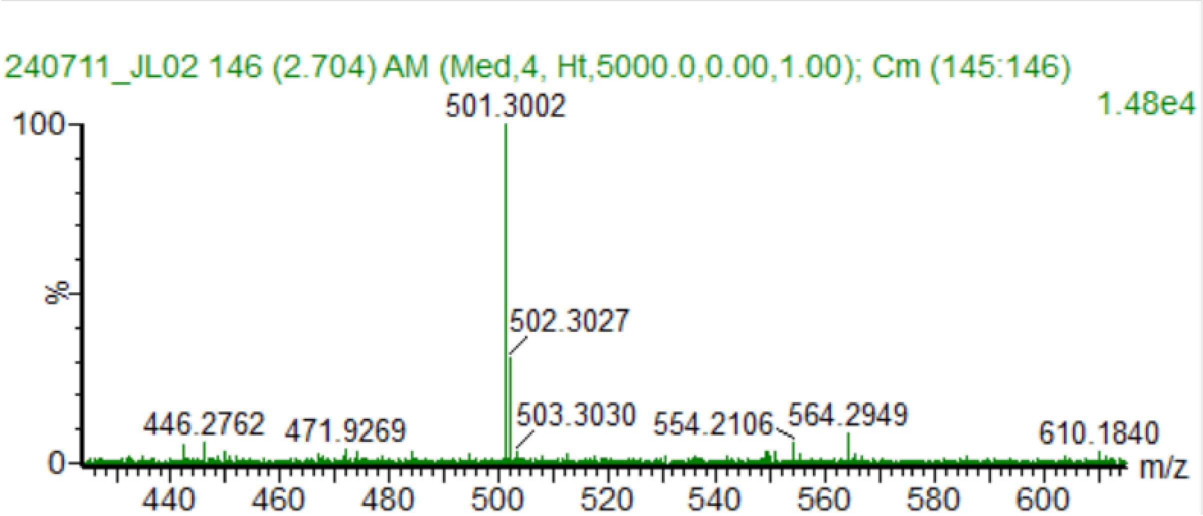

Figure S5-3: HRMS spectrum of Compound **5.**

N-(3-fluorophenyl)-1-{N'-[6-(N-{[N'-(3-fluorophenyl)carbamimidamido]methanimidoyl}amino)hexyl]carbamimidamido}methanimidamide (6)

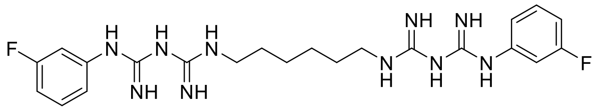

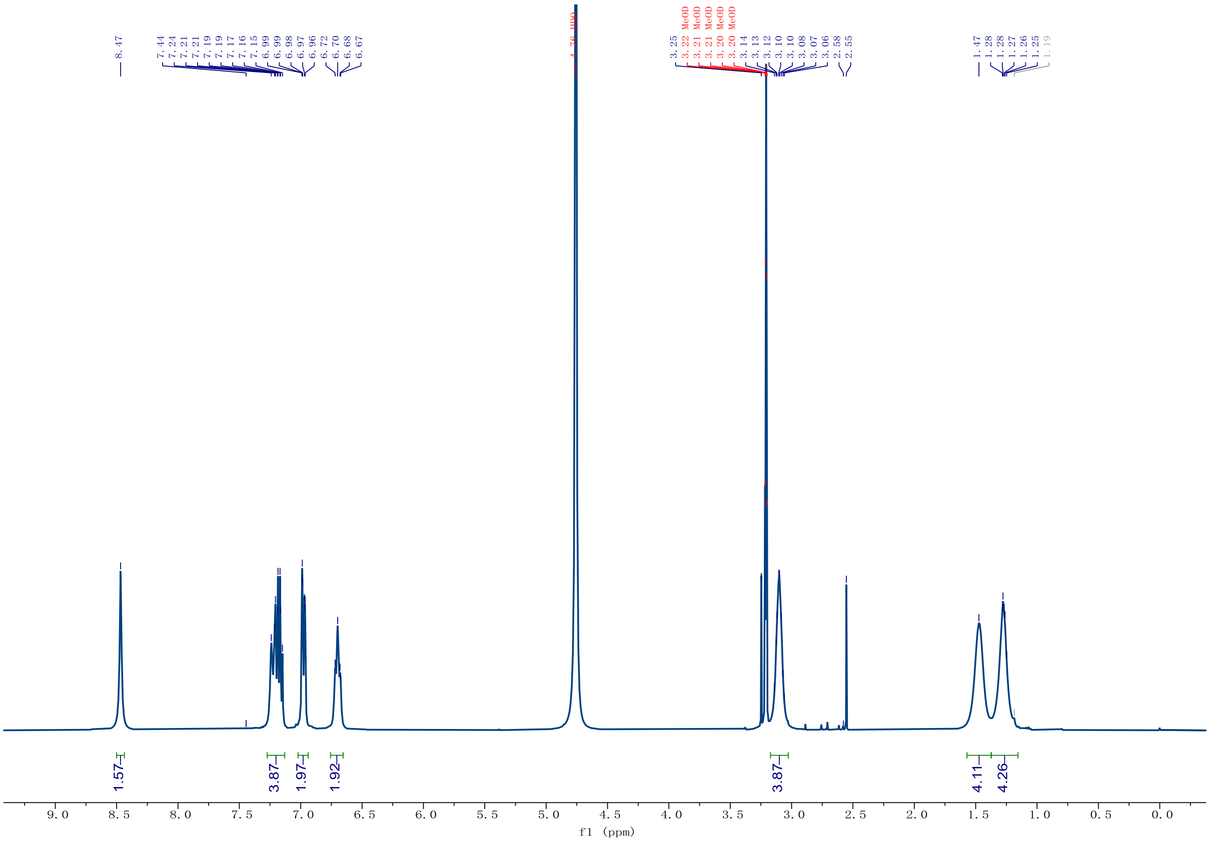

Figure S6-1: Proton ^{1}H NMR spectrum of Compound **6.**

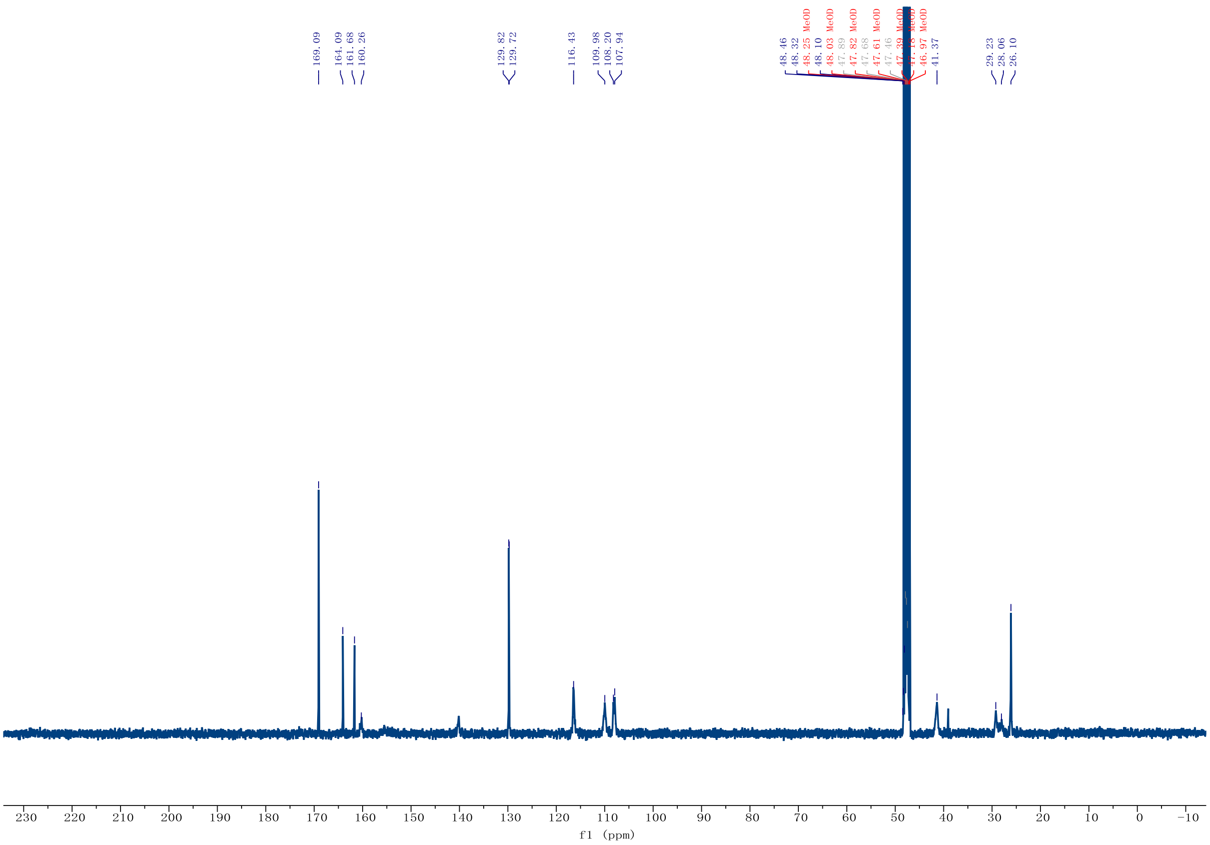

Figure S6-2: Carbon ^{13}C NMR spectrum of Compound **6.**

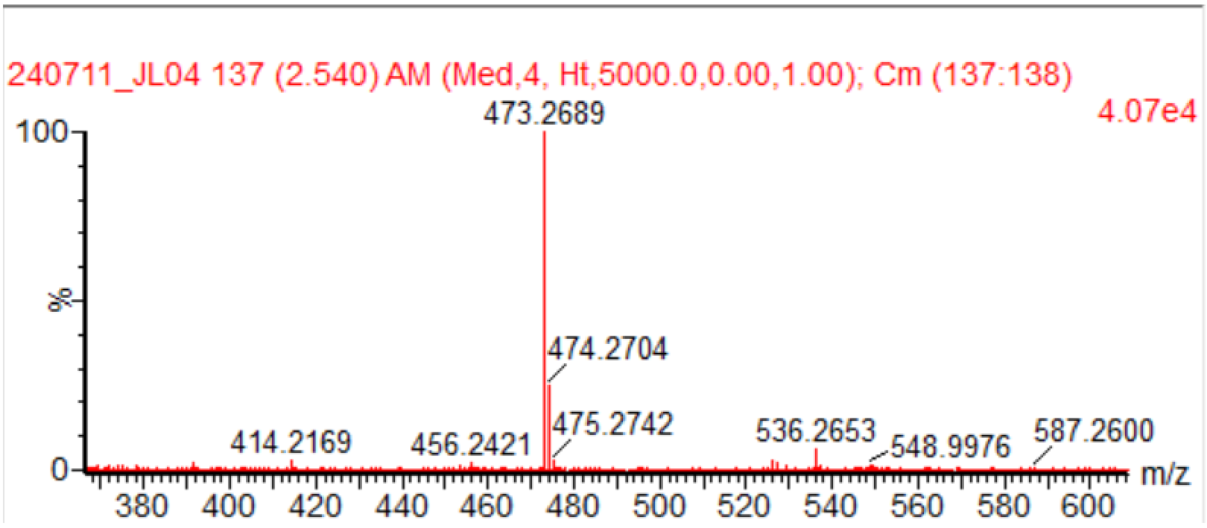

Figure S6-3: HRMS spectrum of Compound **6.**

N-(3,4-difluorophenyl)-1-{N'-[6-(N-{[N'-(3,4-difluorophenyl)carbamimidamido]methanimidoyl}amino)hexyl]carbamimidamido}methanimidamide (7)

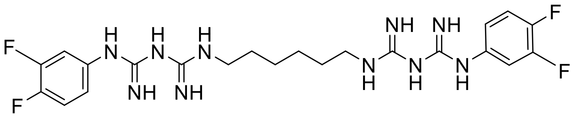

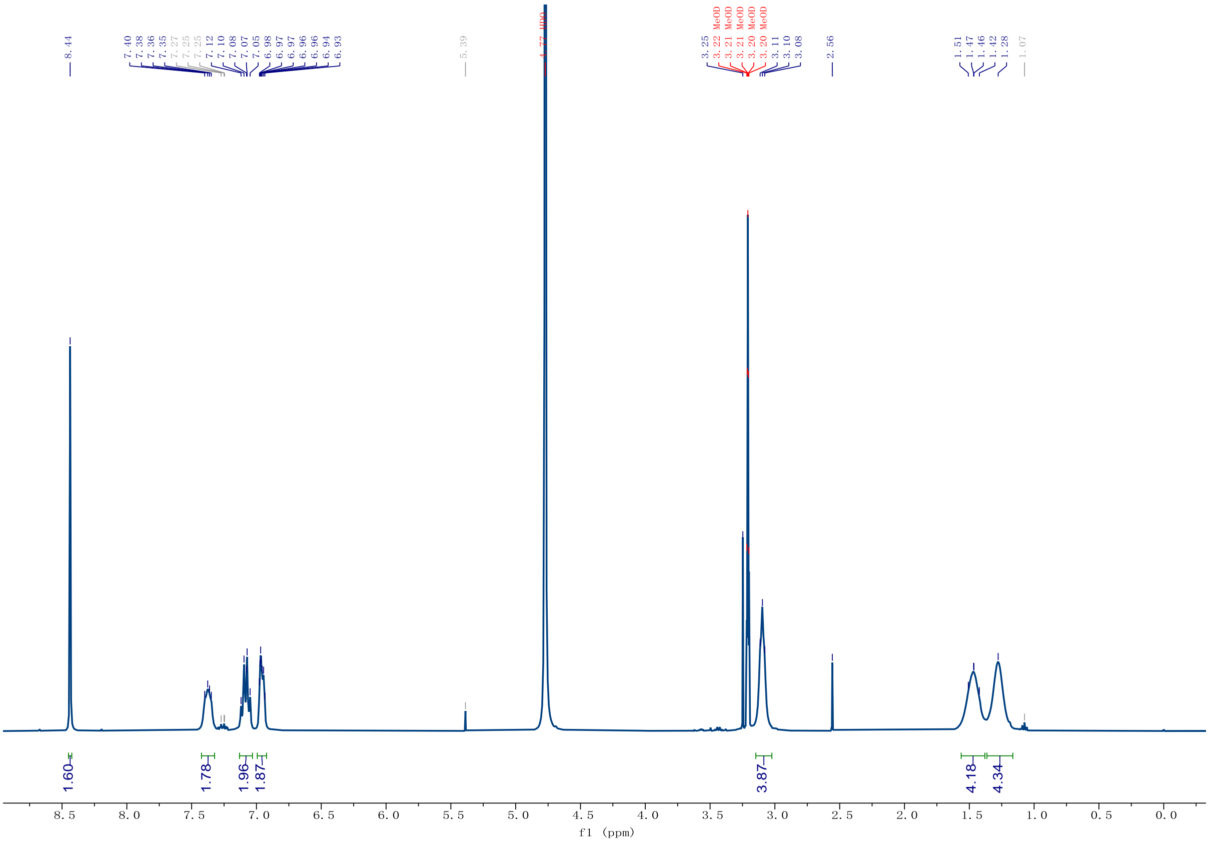

Figure S7-1: Proton ^{1}H NMR spectrum of Compound **7.**

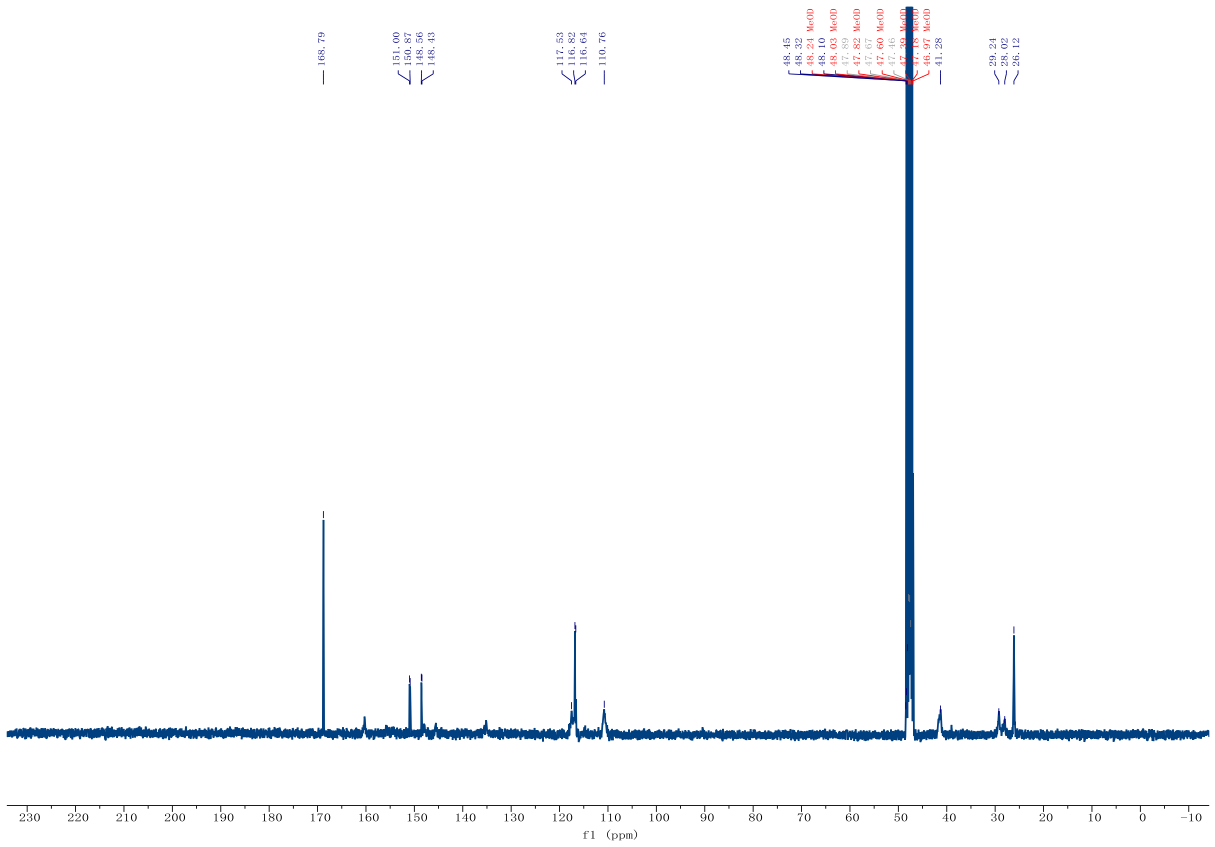

Figure S7-2: Carbon ^{13}C NMR spectrum of Compound **7.**

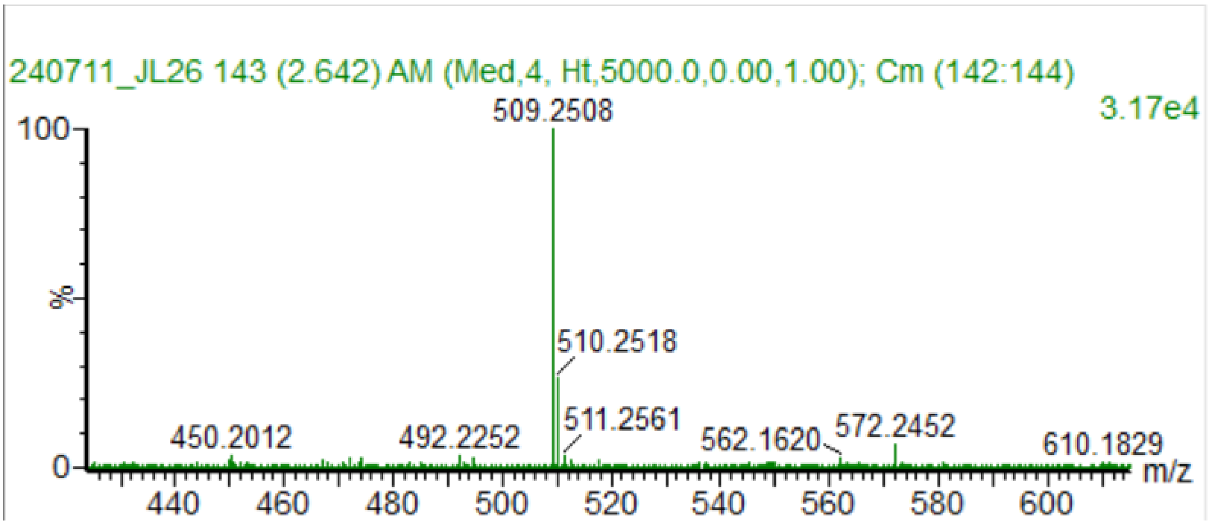

Figure S7-3: HRMS spectrum of Compound **7.**

N-(3-fluoro-4-methylphenyl)-1-{N'-[6-(N-{[N'-(3-fluoro-4-methylphenyl)carbamimidamido]methanimidoyl}amino)hexyl]carbamimidamido}methanimidamide **(8)**

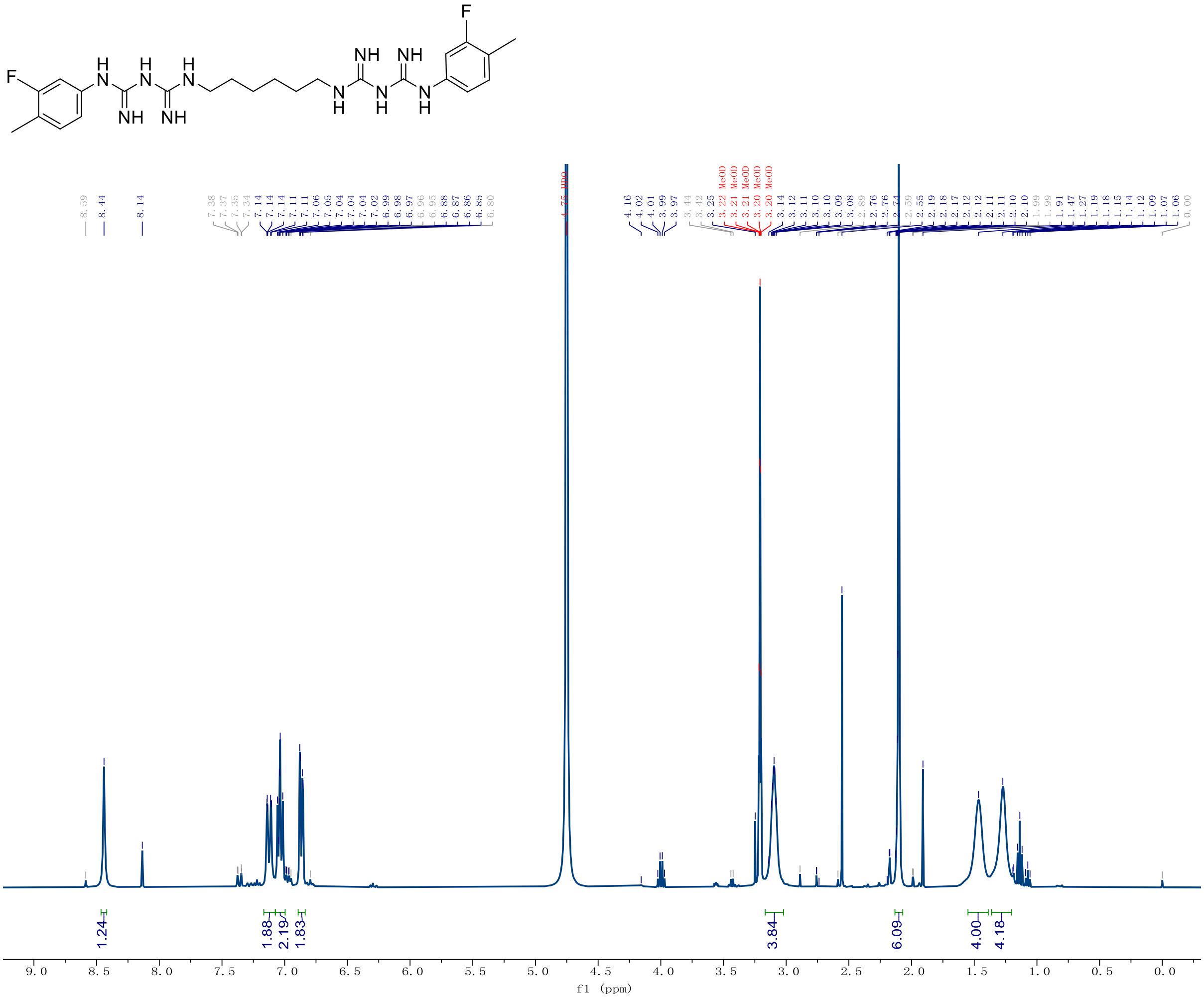

Figure S8-1: Proton ^{1}H NMR spectrum of Compound **8.**

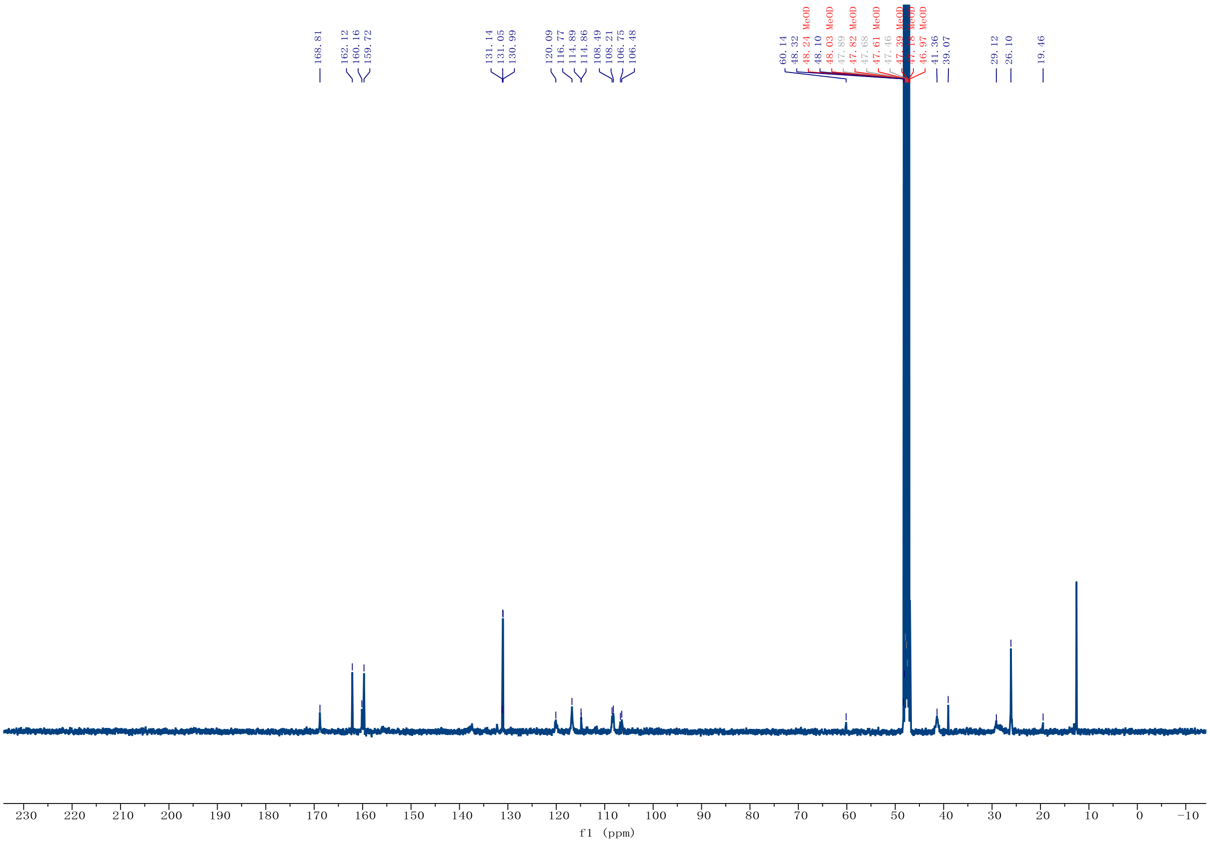

Figure S8-2: Carbon ^{13}C NMR spectrum of Compound **8.**

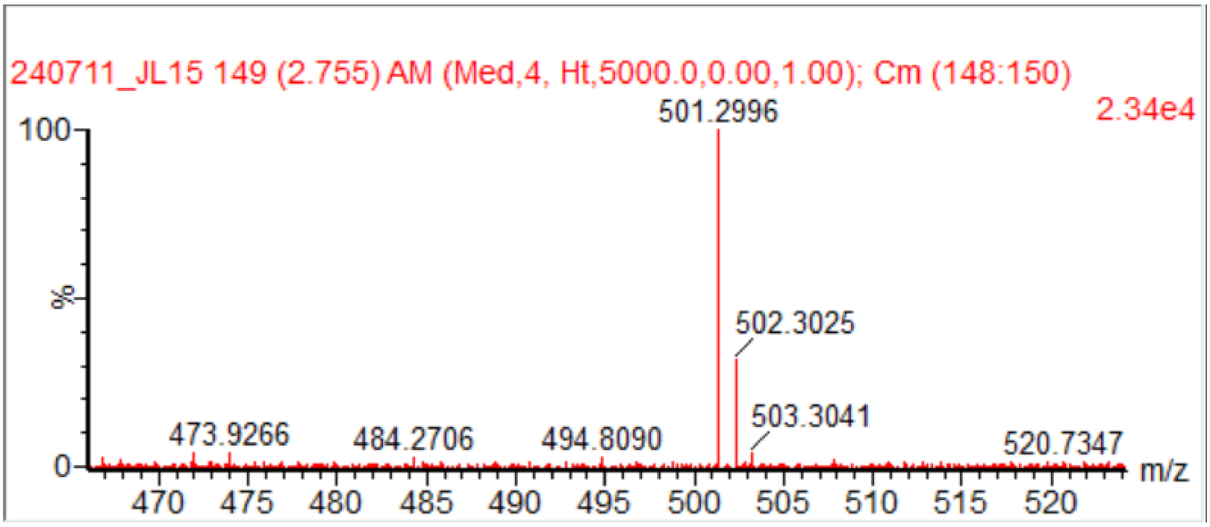

Figure S8-3: HRMS spectrum of Compound **8.**

N-(3-fluoro-4-methoxyphenyl)-1-{N'-[6-(N-{[N'-(3-fluoro-4-methoxyphenyl)carbamimidamido]methanimidoyl}amino)hexyl]carbamimidamido}methanimidamide (9)

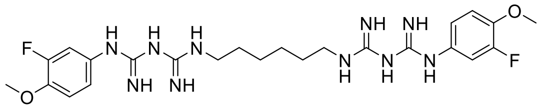

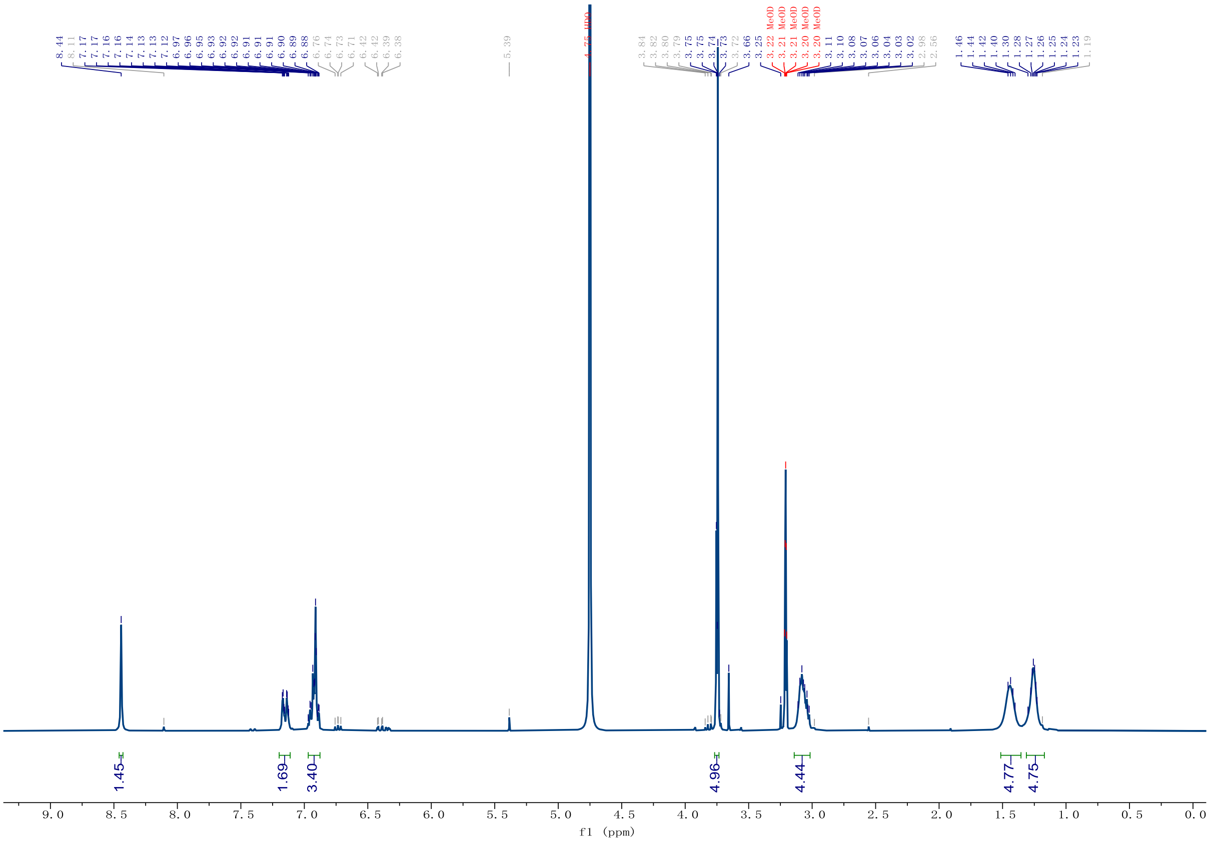

Figure S9-1: Proton ^{1}H NMR spectrum of Compound **9.**

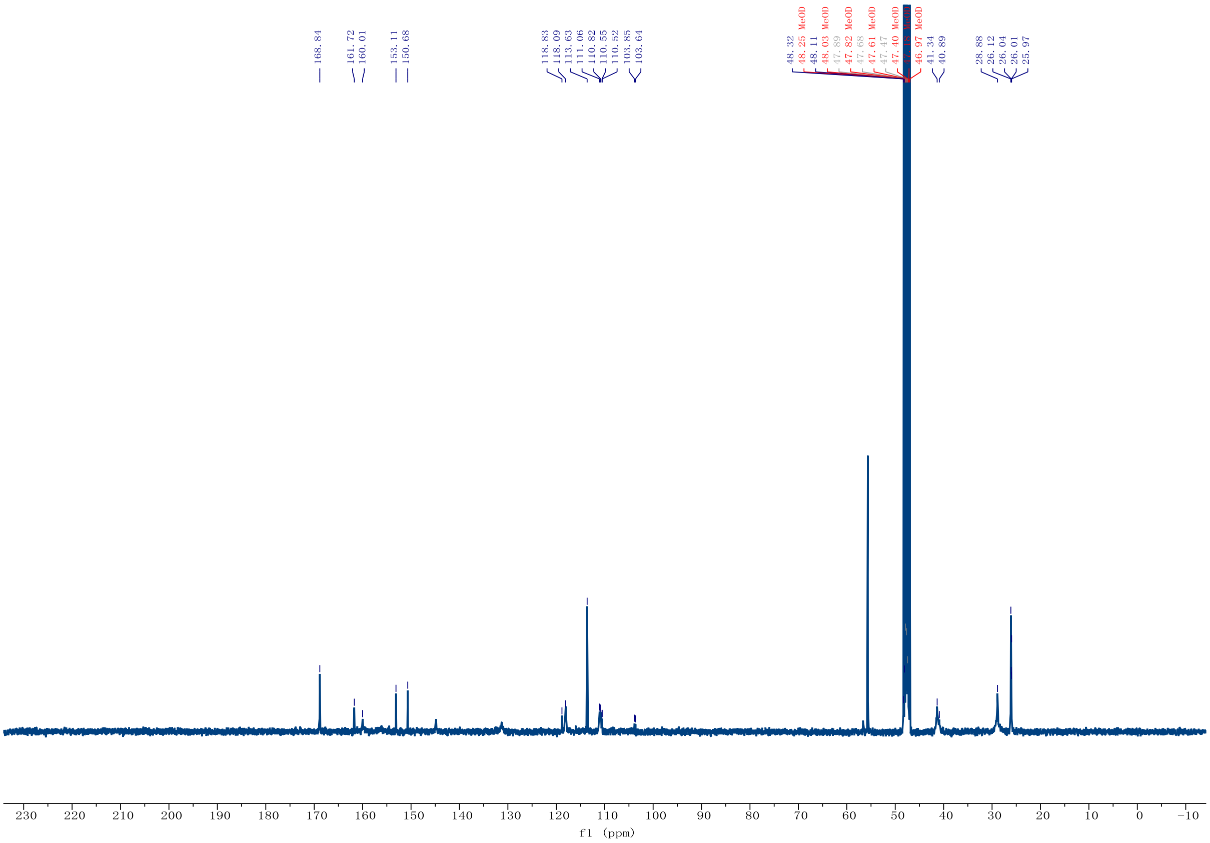

Figure S9-2: Carbon ^{13}C NMR spectrum of Compound **9.**

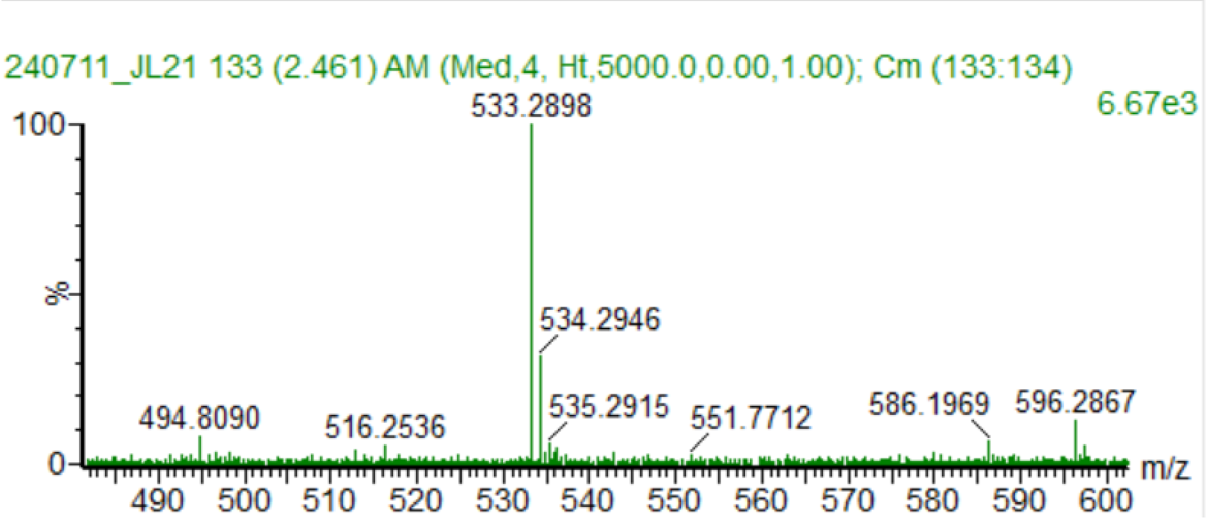

Figure S9-3: HRMS spectrum of Compound **9.**

N-(4-fluoro-3-methoxyphenyl)-1-{N'-[6-(N-{[N'-(4-fluoro-3-methoxyphenyl)carbamimidamido]methanimidoyl}amino)hexyl]carbamimidamido}methanimidamide (10)

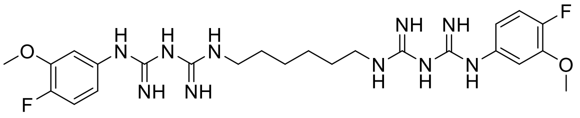

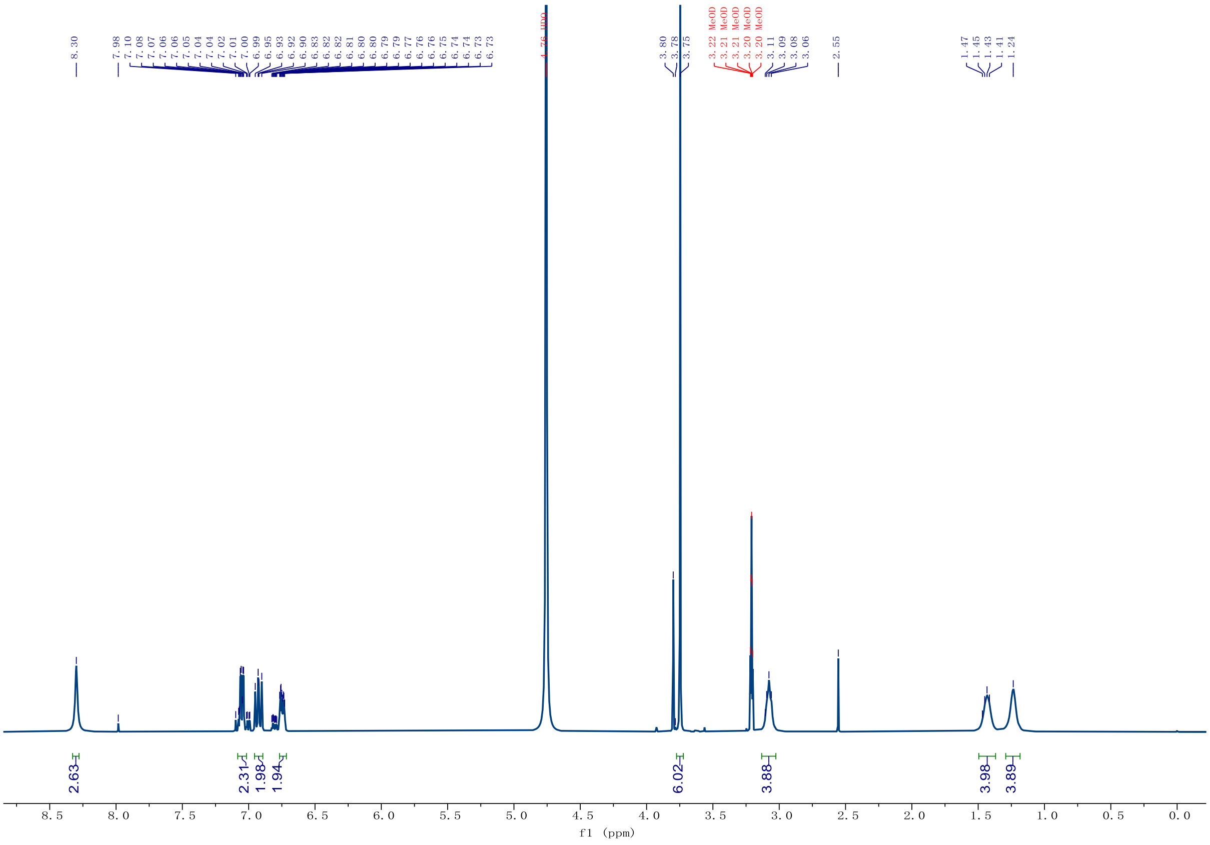

Figure S10-1: Proton ^{1}H NMR spectrum of Compound **10.**

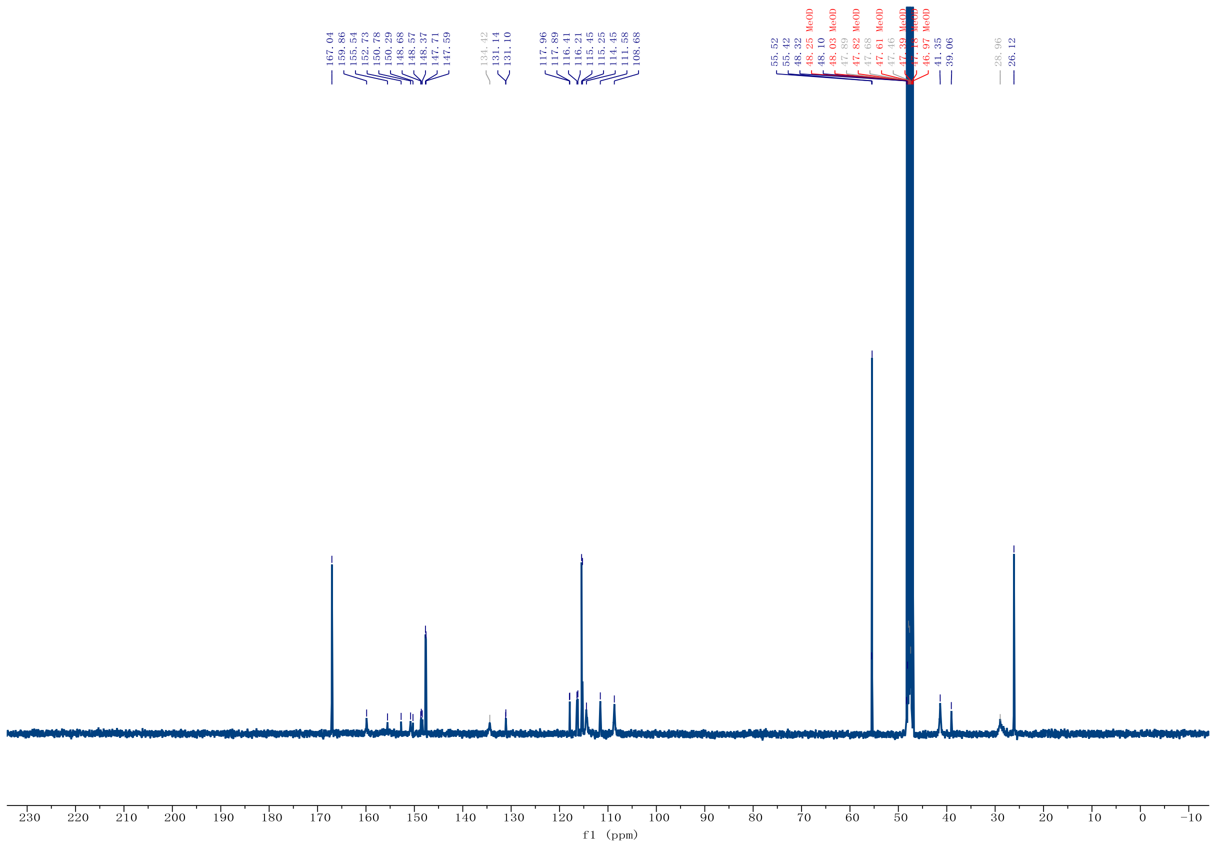

Figure S10-2: Carbon ^{13}C NMR spectrum of Compound **10.**

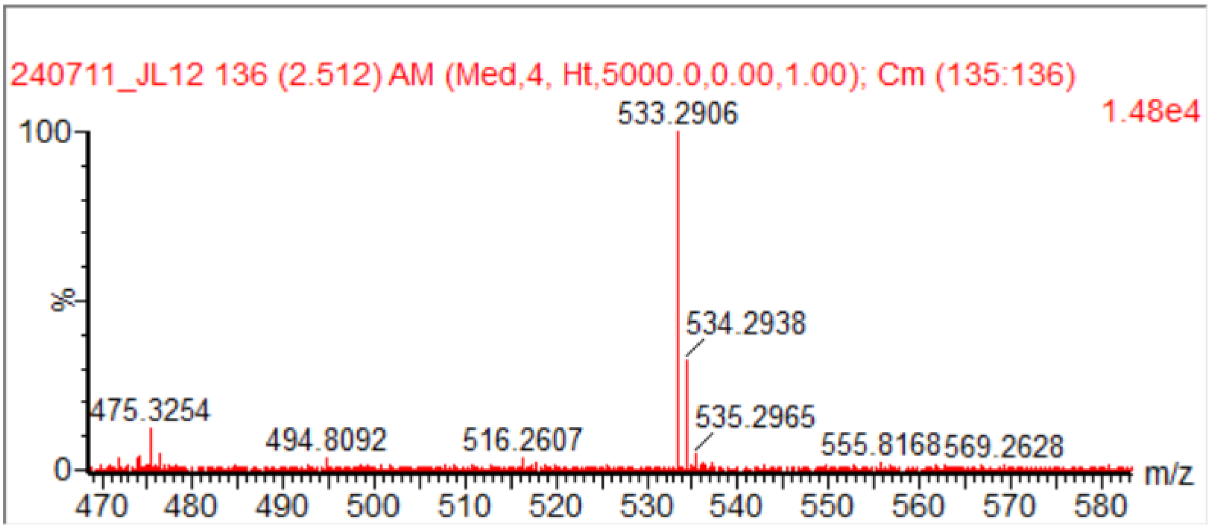

Figure S10-3: HRMS spectrum of Compound **10.**

N-(3,5-fluoro-4- methylphenyl)-1-{N'-[6-(N-{[N'-(3,5-fluoro-4-methylphenyl)carbamimidamido]methanimidoyl}amino)hexyl]carbamimidamido}methanimidamide (11)

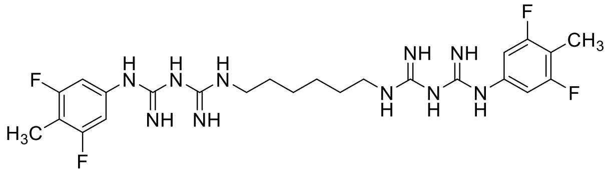

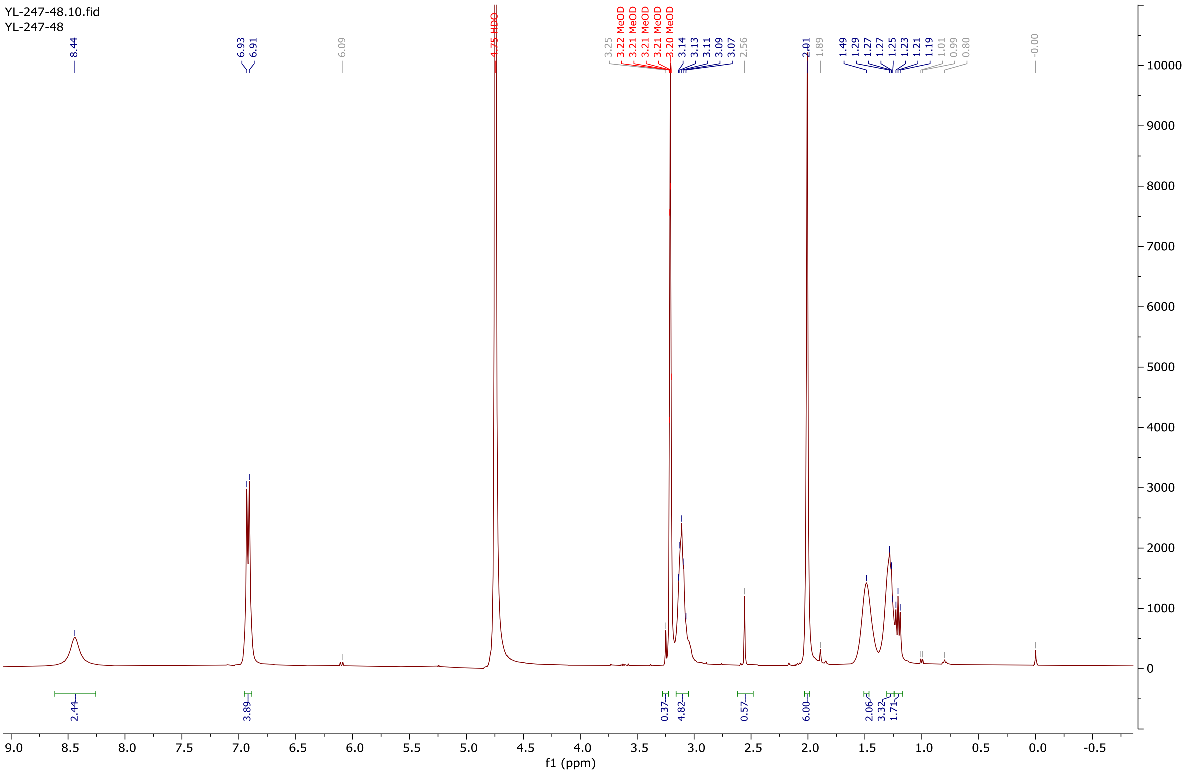

Figure S11-1: Proton ^{1}H NMR spectrum of Compound **11.**

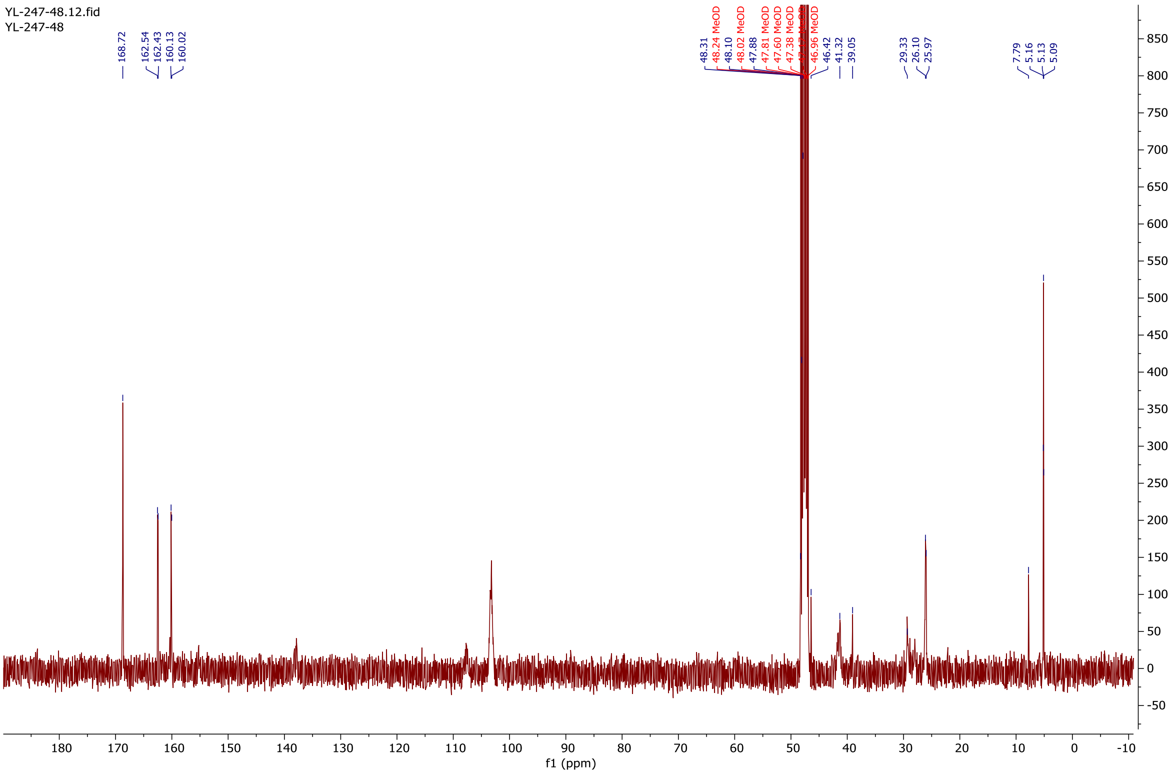

Figure S11-2: Carbon ^{13}C NMR spectrum of Compound **11.**

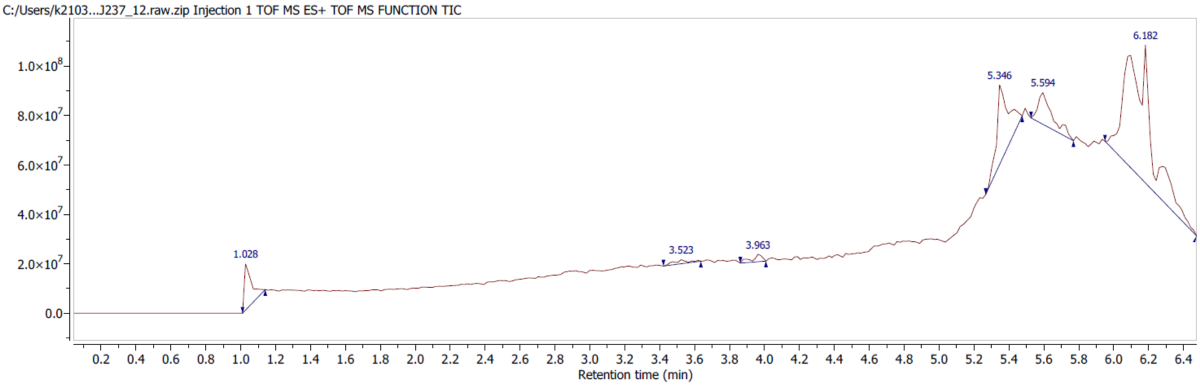

Figure S11-3: HRMS spectrum of Compound **11.**

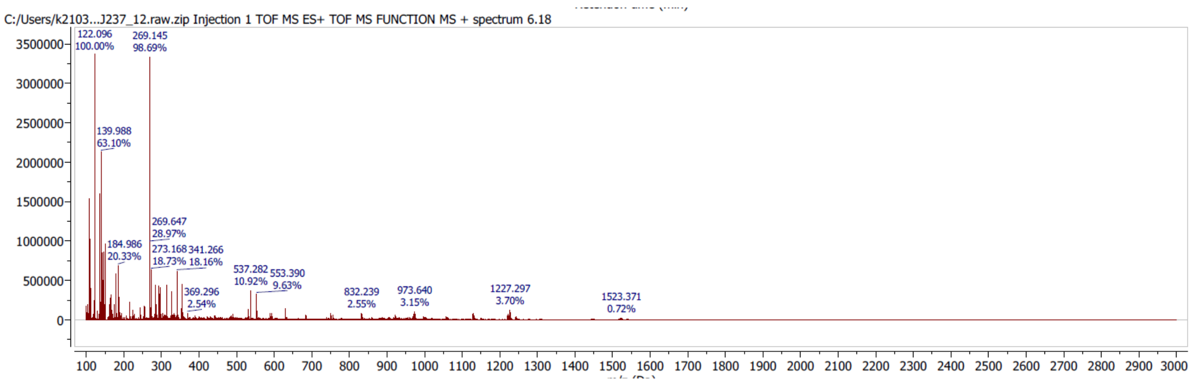

Figure S11-4: HRMS spectrum of Compound **11.**

N-(2,3-fluoro-4- methylphenyl)-1-{N’-[6-(N-{[N’-(2,3-fluoro-4-methylphenyl)carbamimidamido]methanimidoyl}amino)hexyl]carbamimidamido}methanimidamide (12)

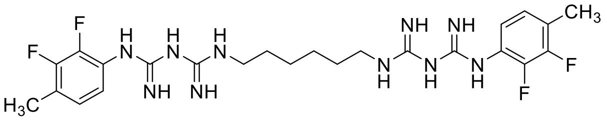

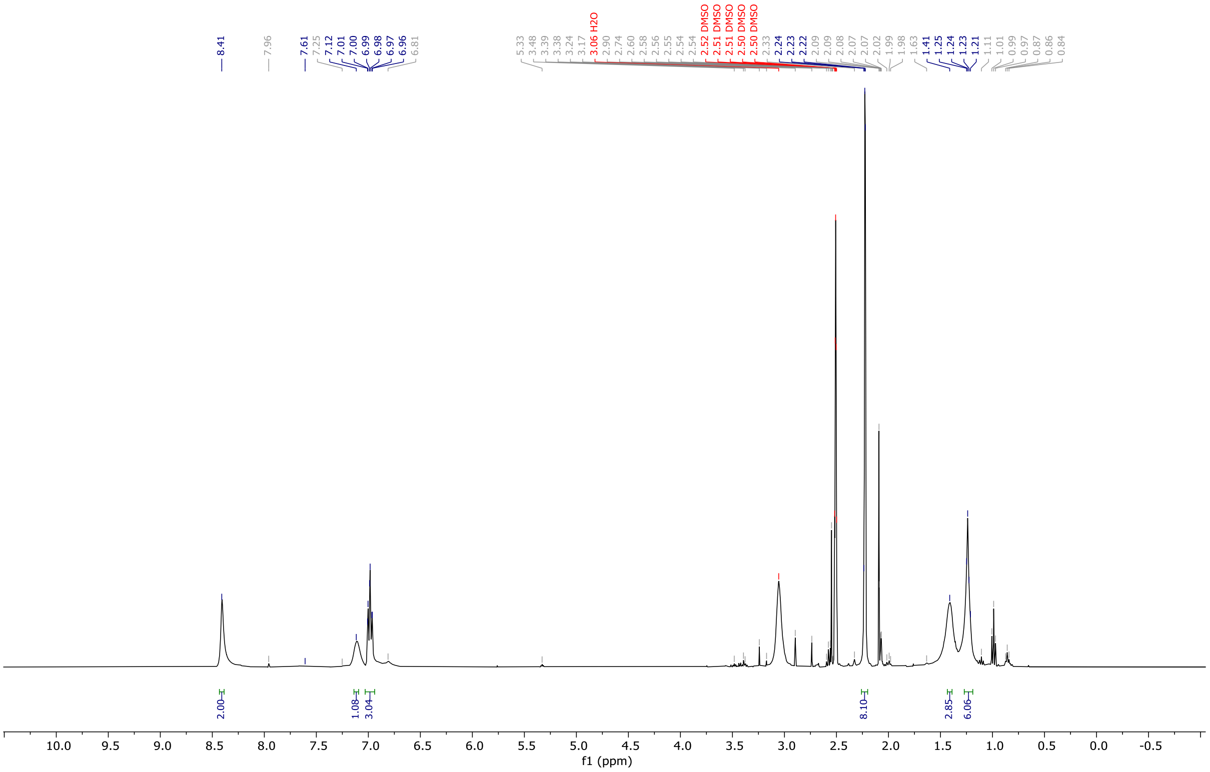

Figure S12-1: Proton ^{1}H NMR spectrum of Compound **12.**

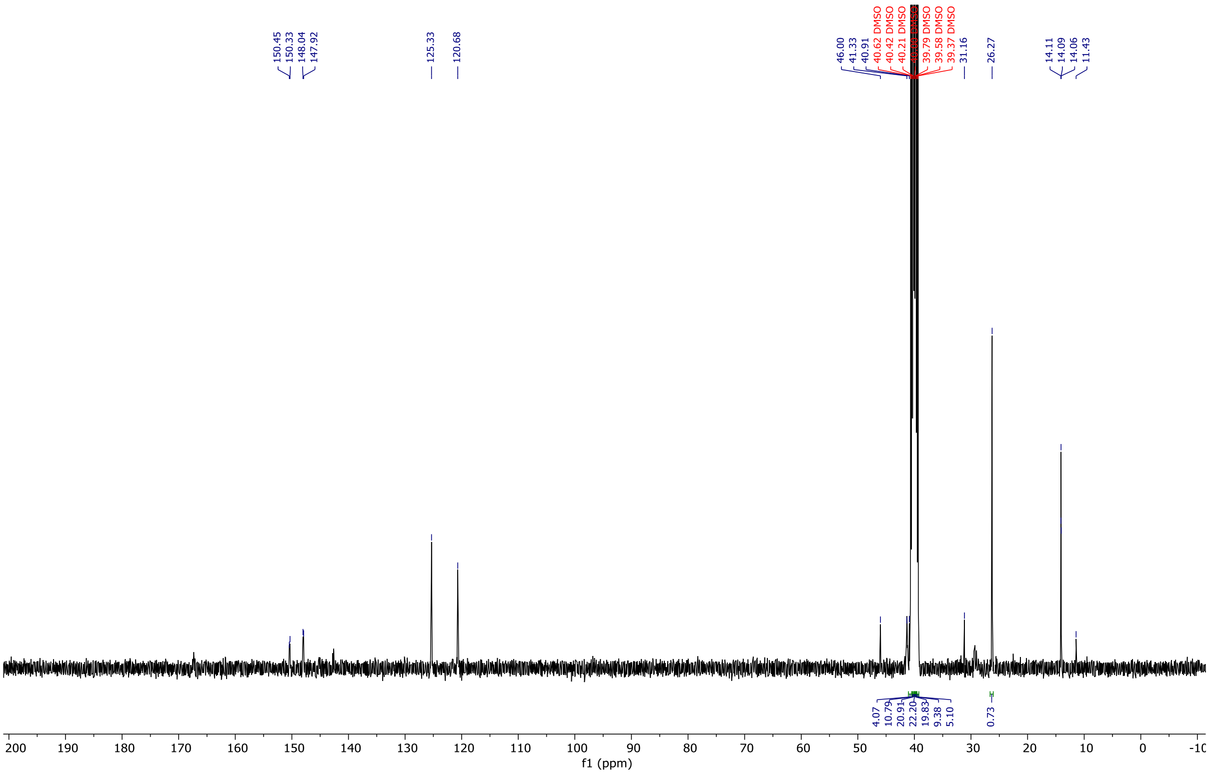

Figure S12-2: Carbon ^{13}C NMR spectrum of Compound **12.**

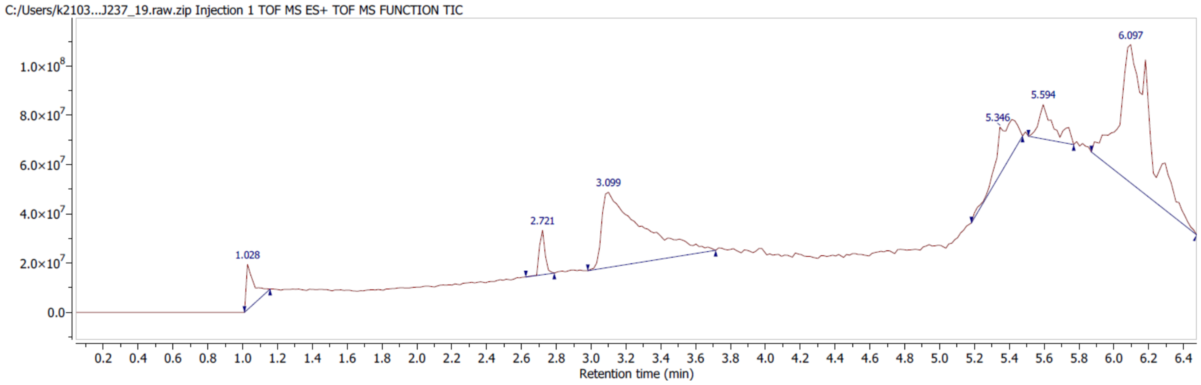

Figure S12-3: HRMS spectrum of Compound **12.**

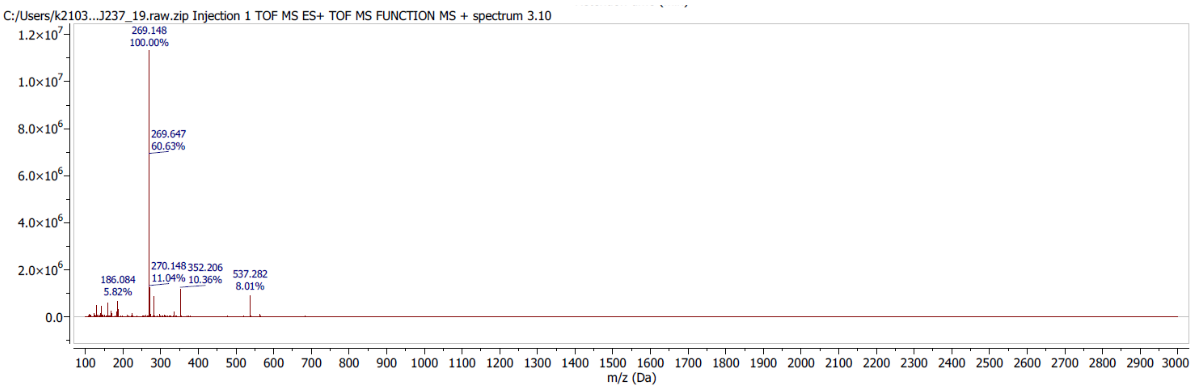

Figure S12-4: HRMS spectrum of Compound **12.**

N-(3-fluoro-5-amine-4- methylphenyl)-1-{N’-[6-(N-{[N’-(3-fluoro-5-amine-4-methylphenyl)carbamimidamido]methanimidoyl}amino)hexyl]carbamimidamido}methanimidamide (13)

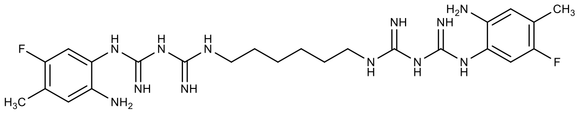

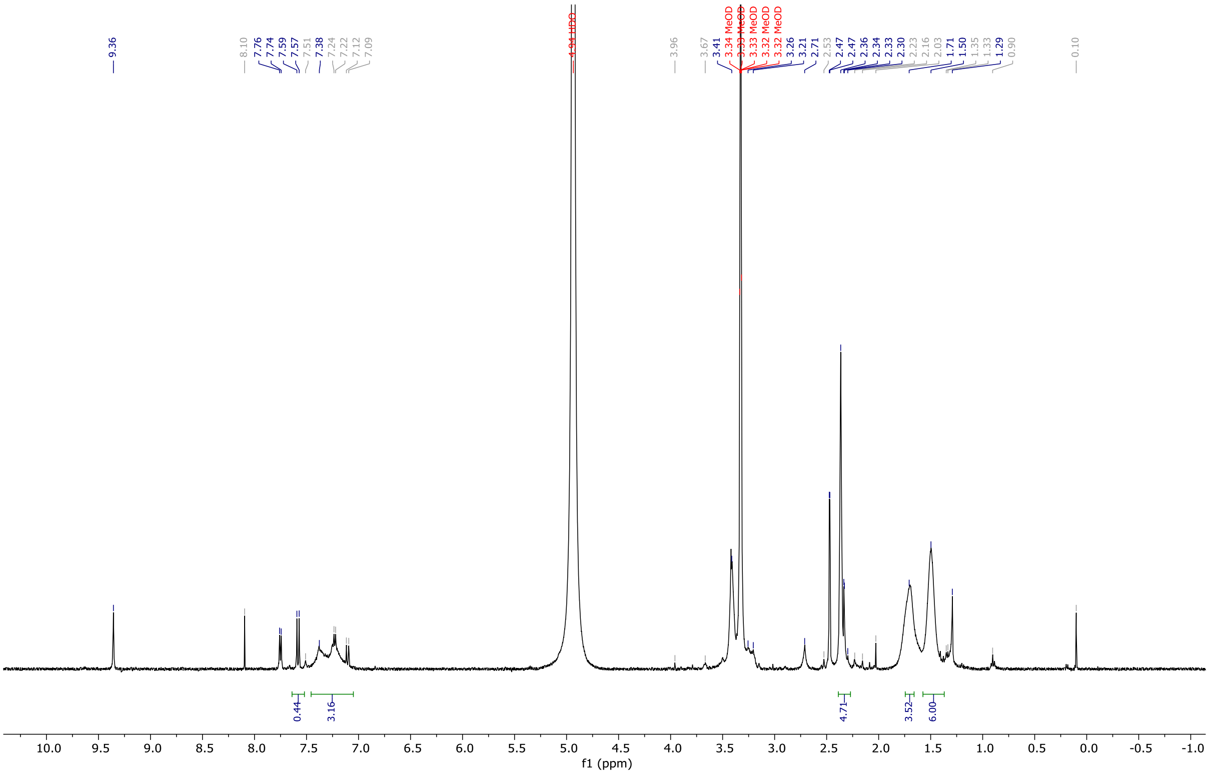

Figure S13-1: Proton ^{1}H NMR spectrum of Compound **13.**

Figure S13-2: Carbon ^{13}C NMR spectrum of Compound **13.**

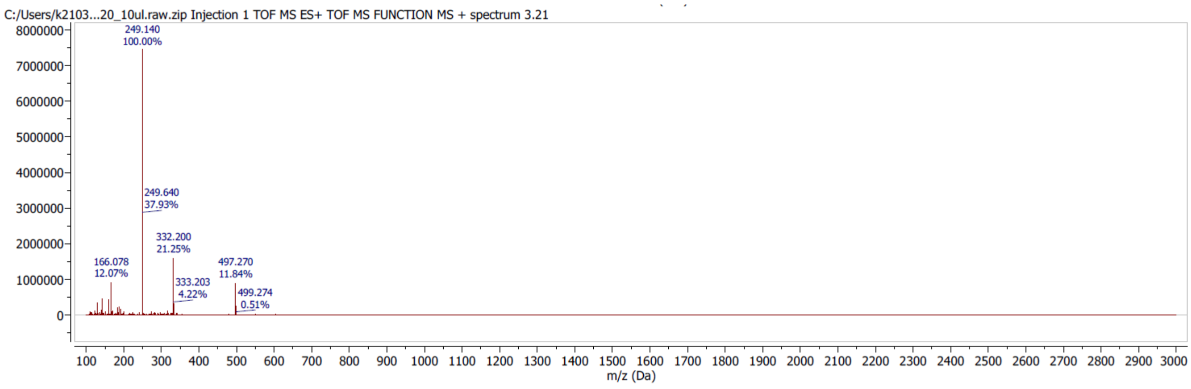

Figure S13-3: HRMS spectrum of Compound **13.**
